# Gut microbes and metabolites as modulators of blood-brain barrier integrity and brain health

**DOI:** 10.1080/19490976.2019.1638722

**Published:** 2019-08-01

**Authors:** Aimée Parker, Sonia Fonseca, Simon R. Carding

**Affiliations:** a Gut Microbes and Health Research Programme, Quadram Institute Bioscience, Norwich, UK; b Norwich Medical School, University of East Anglia, Norwich, UK

**Keywords:** Microbiota, gut-brain axis, metabolites, blood-brain barrier

## Abstract

The human gastrointestinal (gut) microbiota comprises diverse and dynamic populations of bacteria, archaea, viruses, fungi, and protozoa, coexisting in a mutualistic relationship with the host. When intestinal homeostasis is perturbed, the function of the gastrointestinal tract and other organ systems, including the brain, can be compromised. The gut microbiota is proposed to contribute to blood-brain barrier disruption and the pathogenesis of neurodegenerative diseases. While progress is being made, a better understanding of interactions between gut microbes and host cells, and the impact these have on signaling from gut to brain is now required. In this review, we summarise current evidence of the impact gut microbes and their metabolites have on blood-brain barrier integrity and brain function, and the communication networks between the gastrointestinal tract and brain, which they may modulate. We also discuss the potential of microbiota modulation strategies as therapeutic tools for promoting and restoring brain health.

## Introduction

The gut microbiota play an essential role in the functioning of many organs in the body, including the brain. Our understanding of the composition and metabolism of the diverse ecosystem populating the gut, and the complex communication networks comprising the gut-brain axis are rapidly increasing in hand with advances in high-throughput ‘omics-based technologies. This review summarises findings linking the gut microbiota and brain health, with a focus on the impact of gut microbes, and the metabolites they generate from dietary components, on blood-brain barrier integrity. We discuss how these metabolites travel throughout the body along bidirectional neural and circulatory routes, from the perspective of the current knowledge of specific mechanisms. We also highlight the potential therapeutic potential on brain health of manipulating the gut microbiota via delivery of prebiotics, probiotics, and fecal microbiota transplantation. Such approaches can be effective in treating gastrointestinal disorders, and while their suitability and efficacy to treat neurological and neurodegenerative conditions require investigation, they are promising tools that may open unexplored routes to disease treatment and prevention through precise microbiome-targeted therapies.

## The intestinal microbiota in health and neurodegenerative diseases

The human microbiota consists of a diverse and dynamic population of microbes, including bacteria, archaea, viruses, fungi, and protozoa, that establish a mutualistic relationship with the host. More than 10^14^ bacteria cells populate the gastrointestinal (GI) tract the vast majority of which (10^10^–10^12^ CFU/g of intestinal content) are located in the ileum and colon.^1^^2^ A taxonomically diverse intestinal microbiota is associated with integrity of the epithelial barrier, and maintenance of intestinal metabolic and immune homeostasis. Some species of *Lactobacillus* and *Bifidobacterium* genera are considered beneficial, with the presence of *Lactobacillus rhamnosus*, for example, associated with increased intestinal barrier function and expression of epithelial tight junction proteins, and decreased serum levels of inflammatory cytokines.^3^ By comparison, overgrowth of *Clostridium* is often a feature of intestinal microbial dysbiosis and increased intestinal permeability (“leaky gut syndrome”), a feature of inflammatory bowel diseases and other immune-related intestinal and extra-intestinal disorders.^4^ Beyond the GI-tract, the microbiota may also play a role in the functioning of other organ systems including the brain.^5^ Evidence for the GI-microbiota influencing the brain comes primarily from animal studies, in particular germ-free rodents, in which structural and functional attributes of the brain can be assessed prior to, and after, microbial exposure and colonization. The absence of gut microbes is associated with structural alterations in the blood-brain barrier (BBB), characterized by decreased expression of tight junction proteins, leading to increased permeability compared to conventional-specific pathogen free (SPF) mice.^6^ Of note, conventionalizing germ-free mice using fecal material from SPF mice, decreases the permeability of the BBB,^6^ providing evidence of a causal relationship between gut microbes and BBB integrity. Related experiments using chronically-administered antibiotics to deplete the gut microbiota in mice resulted in object recognition impairment^7^ and decreased hippocampal neurogenesis and memory retention, which were reversed by administering probiotic bacteria.

Recent studies suggest the gut microbiota may contribute to the development or progression of neurodegenerative diseases and dementia. Neurodegenerative diseases are characterized by impaired motor function and/or dementia, which is one of the major causes of disability and dependency among older people worldwide.^8^ Dementia is a collection of symptoms used to categorize and diagnose the disease type. Common forms include Alzheimer’s disease (AD), vascular dementia, frontotemporal dementia, Parkinson’s disease (PD), and dementia with Lewy bodies; all are chronic or progressive in nature and feature deterioration in memory, thinking, behavior, and the ability to perform everyday activities. A common feature of all dementias is chronic neuroinflammation, involving overactivation and dysregulation of microglia,^9^ the resident macrophage-like immune cells of the brain. When microglia are activated, their morphology changes in association with increased secretion of pro-inflammatory cytokines, such as IFN-γ, IL-6, and TNF-α, and the release of reactive oxygen and nitrogen species,^10^ which can lead to neuronal cell death, loss of BBB integrity, and brain damage. Whether changes in BBB integrity are a cause or a consequence of neuroinflammation in patients is a major unresolved question.^11^ This central question has been approached using animal models and, in particular, genetically modified mice which develop features of AD or PD. Transgenic mice expressing the amyloid-β precursor protein (APP) develop fewer cerebral β-amyloid plaques when maintained under germ-free conditions compared to conventionalized mice.^12^ Consistent with this finding, and supporting a role for the gut microbiota in the pathogenesis of neuroinflammation in this mouse model, antibiotic administration also limits β-amyloid pathology and neuroinflammation.^13^ Possible links between specific commensal bacteria and neuroinflammation have been identified from microbiota taxonomic profiling in PD patients with alterations in populations of potentially beneficial bacteria (e.g. *Prevotella* genus) and pathogenic bacteria (e.g. members of the Enterobacteriaceae family).^14,15^ Although these findings are intriguing and implicate the gut microbiota in neuroinflammation, they and other related studies have, to date, provided limited mechanistic insight and have been unable to identify the pathways by which gut microbes can influence, directly or indirectly, the BBB.

## The blood-brain barrier

**Figure 1.**
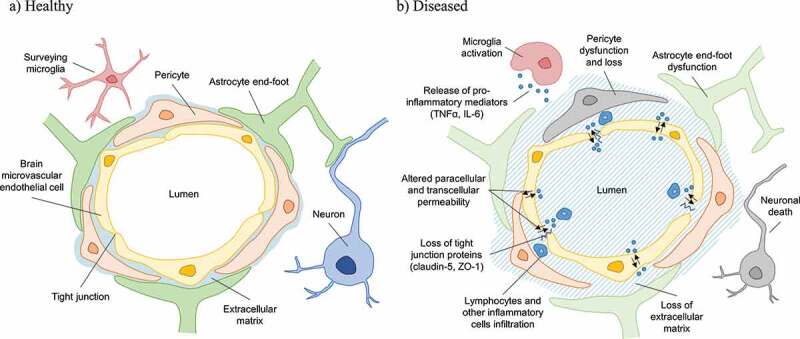
Schematic representation of the human blood-brain-barrier neurovascular-endothelial-unit in health (a) and disease (b) states. Increased permeability related to, for example trauma or infection, is associated with disruption of endothelial tight junctions leading to the ingress and translocation of blood-borne immune cells, and inflammatory mediators such as cytokines and microbes and their products that can activate microglia, resulting in local inflammation leading to loss of extracellular matrix and astrocyte, pericyte and neuronal cell dysfunction and death.

The term “blood-brain barrier” was first coined by Stern and Gautier,^16^ who examined the penetration or exclusion of compounds, such as morphine or India ink, between blood and brain, and proposed a role for the barrier in maintaining brain homeostasis. Later studies in the 1960s demonstrated that the BBB was located in the endothelium forming the walls of vessels in the brain.^17,18^ Since these pioneering studies, the concept of the BBB as a static, impermeable barrier has evolved into the current view of a dynamic, highly regulated, specific cellular system,^19^ with increasing awareness of the contribution multiple cell types play in regulating dynamic BBB responses. The BBB is now considered to be part of a neurovascular unit (NVU) (Figure 1) comprising brain microvascular endothelial cells (BMEC), pericytes, astrocytes, neurons, microglia, and extracellular matrix (ECM),^20^ which together contribute to regulating BBB stability and function. This organization is found in all brain regions, except for the circumventricular organs, which regulate the autonomic nervous system and endocrine glands and have fenestrations allowing the diffusion of molecules across vessel walls.^21^

BMECs are interconnected by protein complexes consisting of highly electrically-resistant tight junctions (*zonulae occludens*), which limit the paracellular flow of molecules between adjacent endothelial cells to maintain ionic homeostasis in the brain.^22^ Joining the BMECs are several non-endothelial cells including pericytes that wrap around the capillary endothelium^23^ and can modify vascular diameter and regulate transcytosis and immune cell trafficking across the BBB.^24^ Astrocytes and their endfeet form a second physical, transport, and metabolic barrier around pericytes and endothelial cells, and communicate with neurons to establish an endothelium-neuron link.^25^ Pericytes and astrocytes also transmit neuronal signals to the local vasculature, affecting barrier physiology by altering arteriolar dilation and blood flow.^26^ While implicated in the regulation of vascular growth during angiogenesis, the role of microglia in homeostatic BBB maintenance is not clear, but these cells are major players in the inflammation associated with neurodegenerative diseases, secreting pro-inflammatory cytokines and factors promoting BBB breakdown and neuronal loss. In inflammatory states, remodeling of BMEC tight junctions increases transcellular permeability, allowing circulating inflammatory cytokines and effectors into the brain. Inflammatory activation of vascular endothelium can induce BMEC expression of adhesion molecules and chemoattractants, allowing infiltration of neutrophils and effector Th1 and Th17 subsets of CD4^+^ T cells which are implicated in the pathogenesis of multiple sclerosis (MS), AD and PD.^27,28^ Astrocyte morphology and secretion of inflammatory cytokines and chemoattractants are altered in early stages of AD pathology.^26^ Amyloid-β protein can induce BMEC activation and tight junction remodeling, and pericyte damage and loss leads to cognitive impairment, increased amyloid-β deposition, and tau pathology.^29^ In summary, a combination of inflammation and changes in BBB component cell functions contribute to neurodegenerative disease-associated BBB breakdown. Infiltrating immune cells, microglial cells, and astrocytes may reciprocally activate each other, driving chronic inflammation and preventing BBB repair, with pericyte damage and neuronal loss further promoting cognitive decline. Preserving and restoring BBB integrity therefore presents a primary target for developing neuroprotective strategies and interventions.

## Gut microbiota-derived metabolites influencing BBB integrity and brain health

The gut microbiota transform dietary components, including macro- and micronutrients, fibs, and polyphenols, into a range of metabolites, including short-chain fatty acids, trimethylamines, amino acid derivatives, and vitamins. These microbial-derived metabolites and dietary components have essential metabolic and signaling functions which can modulate host homeostasis, including BBB integrity and brain function. Microbial metabolites affecting the BBB and brain are detailed below, and specific studies describing effects of some of these metabolites on BBB permeability are summarised in Table 1.

**Table 1.** Effects of gut microbes and their metabolites on blood-brain barrier permeability.

Method	Permeability modulator	Model	Effects on BBB	Reference
*In vivo*	*Clostridium butyricum*	Traumatic brain injury on C57Bl/6 mice	↑ TJ proteins expression ↓ Permeability	^30^
	*Clostridium tyrobutyricum*, *Bacteroides thetaiotaomicron* and sodium butyrate	Germ-free C57Bl/6 mice	↑ TJ proteins expression ↓ Permeability	^6^
	Sodium butyrate	Traumatic brain injury on C57Bl/6 mice	↑ TJ proteins expression ↓ Permeability	^31^
	Acetate/propionate/butyrate	α-synuclein overexpressing germ-free BDF1 mice	↑ Microglia activation	^15^
	Chenodeoxycholic acid and deoxycholic acid	Bile duct ligation on Sprague Dawley rats	↑ Permeability	^32^
*In vitro*	Propionate	hCMEC/D3 treated with LPS	↓ Paracellular permeability ↑ TEER	^33^
	Chenodeoxycholic acid and deoxycholic acid	RBMECs	↑ Permeability	^32^

***Short Chain Fatty Acids (SCFA***): are small organic monocarboxylic acids, principally produced by colonic fermentation of dietary fib and complex plant-based polysaccharides, to generate energy and release carbon, permitting microbial growth. SCFAs are present at an approximate concentration of 100 mM in the colonic lumen at a ratio of 60:25:15 for acetate:propionate:butyrate, respectively.^34^ The production of propionate is primarily restricted to members of the Clostridiales order of anaerobic bacteria, with production of acetate and butyrate being more broadly distributed.^35^ Over 95% of SCFAs produced in the colon are absorbed by the mucosa of healthy individuals^36^ with all three detectable in the peripheral blood at ratios different to those in the lumen (acetate, 22–42 μM; propionate, 0.9–1.2 μM; butyrate, 0.3–1.5 μM).^33^ SCFAs stimulate colonic blood flow, upper-gut motility, influence water and salt uptake, and enhance satiety.^36^ They can also access the BBB via the bloodstream to impact directly on its integrity.^37^ For example, BBB permeability decreases in germfree mice after colonization with the butyrate producer *Clostridium tyrobutyricum* or, after oral administration of sodium butyrate, by upregulating tight junction proteins.^6^ Following traumatic brain injury, intravenous or intraperitoneal administration of sodium butyrate can prevent BBB breakdown and promote neurogenesis.^30,31,38^ It is therefore conceivable that modulating SCFA levels could be useful in preventing or treating neural decline. Intraperitoneal administration of butyrate or increased dietary soluble fib (inulin) intake improve neuroinflammation in aging mice,^39^ and in CK-p25 transgenic mice, which exhibit synaptic and neuronal loss and learning, intracerebroventricular injections of butyrate modify histone acetylation in the hippocampus to improve memory and learning.^40^ Protective, anti-inflammatory, and permeability-reducing effects of propionate treatment have also been described in a human brain endothelial cell culture model.^33^ On the other hand, high brain levels of propionate can exacerbate symptoms in a mouse model of autism, but this can be mitigated with butyrate supplementation,^41^ suggesting metabolite balance rather than individual metabolite levels may be important. The maturation of microglial cells, which regulate BBB integrity, neuroinflammation, and neurogenesis, is modified by SCFA in germfree mice, where the reduced microglia number, function and morphology can be rescued by 4-week delivery of an SCFA cocktail in drinking water.^42^ Pending clinical trials on specific-optimized SCFA supplementations, it has been hypothesized that providing SCFA mixtures or simply increasing dietary fib could rescue the hypometabolism associated with neuronal dysfunction in neurodegenerative conditions including AD.^43^ Intriguingly, a handful of recent studies in which bacterial, viral, and fungal-derived nucleic acids and proteins have been detected in post-mortem brains of AD sufferers^44,45^ suggest microbial metabolites such as SCFAs could be produced locally, by brain-resident or infiltrating microbes. The possibility of a ‘brain microbiome’ remains controversial, but while consideration of possible contamination issues is needed in interpreting the detection of nucleic acids from low-abundance microbes in tissue samples, Carrasco and colleagues^46-48^ provide supporting immunostaining data for various fungal species in post-mortem brain tissue from Alzheimer’s patients. These included *Candida albicans,* which is commonly found in the mammalian gut but more rarely isolated from the environment,^49,50^ and the detection of both budding and hyphal forms,^47,48^ indicative of fungal growth within the living tissue. Whilst gut microbes can dictate the metabolite availability within the intestine and the brain via the circulation, whether they have a significant impact on SCFA availability within the brain as ‘local’ residents remains to be determined. Additional work is now needed to understand the optimum balance of SCFAs in the periphery and the brain which promote healthy aging, and further still how the microbiome of the gut (and potentially the brain) can be manipulated to achieve this balance and protect cognitive health.

***Trimethylamines***: are metabolites produced from gut microbial metabolism of dietary choline, lecithin, carnitine and trimethylamine-N-oxide (TMAO) that are present in foods such as eggs, nuts, dairy products, meat, and fish. Choline is degraded into trimethylamine (TMA), which is converted in the liver by flavin-containing monooxygenases (FMOs) into TMAO, and demethylated into dimethylamine and methylamine.^51^ Gut-derived TMA is generated by bacteria of the genera *Anaerococcus, Clostridium, Escherichia, Proteus, Providencia*, and *Edwardsiella*.^52^ The presence of TMAO in human brains indicates its ability to cross the BBB.^53^ TMAs have been associated with both beneficial and detrimental health effects. High plasma levels of TMAO have been associated with increased risk of colorectal cancer^54^ and with risk of developing atherosclerosis and cardiovascular disease via effects on cholesterol metabolism.^55^ This is of interest regarding neurodegenerative diseases including AD and vascular dementia, in which cardiovascular disease and altered cholesterol metabolism are strongly associated with increased risk. In individuals with a hereditary defect in FMO3, bacterial TMA production contributes to the symptoms of trimethylaminuria (TMAU) or fish-odor syndrome.^56^ Therapy with archaebiotics and attempting to modulate the gut microbiota by administering specific strains of TMA metabolizing Archaea has been proposed as a treatment for cardiovascular diseases and TMAU. The methanogen, *Methanomassiliicoccales* can reduce TMA concentration in the gut by converting it to methane, thus decreasing the production of TMAO from TMA in the liver.^57,58^ TMAO’s beneficial effects include reducing endoplasmic reticulum stress and lipogenesis in adipocytes, increasing insulin secretion in pancreatic islets, and attenuating diet-induced impaired glucose tolerance.^59,60^ Again, by extension, such therapies may also be beneficial in protecting against neurodegenerative disease as diabetes which is another dementia-associated risk-factor. More specific to AD, TMAO has also been shown to restore the ability of mutant tau protein to promote microtubule assembly^61,62^ with microtubule disassembly and neuron death being hallmark pathological features of AD.^63^ In addition to its potential use as an AD biomarker,^64^ TMAO may also have a therapeutic effect in AD and other protein-misfolding conditions, by preferentially hydrating partially denatured proteins to correct folding defects and entropically stabilizing native conformations.^65,66^

***Amino acid metabolites***: the gut microbiota plays an essential role in amino acid catabolism the products of which can influence the balance between the production of excitatory and inhibitory neurotransmitters essential for correct brain functioning.^67^ Species of the genera *Lactobacillus* and *Bifidobacterium* metabolize glutamate, the most abundant free amino acid and excitatory neurotransmitter in the brain, to produce γ-aminobutyric acid (GABA), a major inhibitory neurotransmitter.^68^ Decarboxylases secreted by *Clostridium sporogenes* contribute to converting tryptophan, an essential amino acid found in a wide range of foods,^69^ to the neurotransmitter tryptamine, involved in the release of serotonin by cells of the enteroendocrine and enteric nervous systems.^70,71^ Germfree mice display increased plasma tryptophan levels, which are normalized after conventionalization.^72^ *Bifidobacterium infantis* can increase plasma levels of tryptophan.^73^ Bacterial conversion of tryptophan to indoles, ligands for aryl hydrocarbon receptor (AHR), can decrease brain inflammation and limit disease severity in murine models of MS by activating astrocytes.^74^ Tryptophan is a precursor of many other diverse microbial and host metabolites^75^ including kynurenic acid, which has anti-inflammatory properties in the gastrointestinal tract^76^ and is considered to be neuroprotective,^77^ and quinolinic acid, which is a neurotoxin and a BBB modulator implicated in the etiology of psychiatric disorders and neurodegenerative diseases.^78,79^ Tryptophan and glutamate-metabolizing microbes may therefore perform important BBB and brain-protective functions, but greater understanding and care are needed to harness their therapeutic potential while avoiding the effects of potentially harmful derivatives.

***Vitamins***: are essential micronutrients which humans must obtain from their diet and intestinal microbiota. Vitamin K is produced by *Escherichia coli, Klebsiella pneumoniae, Propionibacterium*, and *Eubacterium*; B2 (riboflavin) by *Bacillus subtilis* and *E. coli*; B9 (folic acid) by *Bifidobacterium, Lactococcus lactis* and *Streptococcus thermophilus*; and B12 (cobalamin) by *Lactobacillus reuteri* and *Propionibacterium freudenreichii*.^80^ The uptake of microbe-derived vitamins occurs predominantly in the colon, in contrast to dietary vitamins, which are absorbed in the small intestine.^81^ Vitamins have a variety of roles in maintaining and protecting intestinal and systemic health throughout life. Vitamin K, essential for the process of thrombosis,^82^ is routinely administered to neonates shortly after birth to prevent hemorrhagic disease, prior to the establishment of their gut microbiota.^83^ Low levels of vitamin K have been correlated with apolipoprotein Eϵ4 allele, a risk factor linked to AD.^84^ Vitamin K has also been shown to have a beneficial role in the modulation of α-synuclein fibrillization, associated with PD.^85^ Increased dietary vitamin K intake has been associated with less severe subjective memory complaints in the elderly.^86^ Vitamin B synthesis increases as the intestinal microbiota are being established^87^ with deficiencies leading to serious neurological disorders including beri-beri, polyneuropathy and cerebellar ataxia.^88^ Vitamin B9 has a preventive role in the development of neural tube defects, coronary heart disease, cancer, and neuropsychiatric disorders.^89^ Folate deficiency has been associated with depression, with methylfolate being one of the only medical foods licensed for treating depression.^90,91^ Folate intake below the Recommended Daily Allowance may also increase the risk for mild cognitive impairment and probable dementia among older women.^92^ Similarly, high-dose B-vitamin treatment, including B6, B9, and B12, can decrease levels of homocysteine, a by-product of vitamin B, and slow the atrophy of specific regions of the brain associated with cognitive decline in AD.^93^

In summary, SCFAs and other microbial metabolites can act at the intestinal epithelial barrier, the blood-brain-barrier, directly on brain neurons, or by regulating the endocrine and immune systems to protect against the pathology and inflammation associated with ageing and disease, and may also protect against neurodegeneration by limiting the transit and influx of pathological microbes and their products into the brain.

## Pathways of communication along the microbiota-gut-brain axis

**Figure 2.**
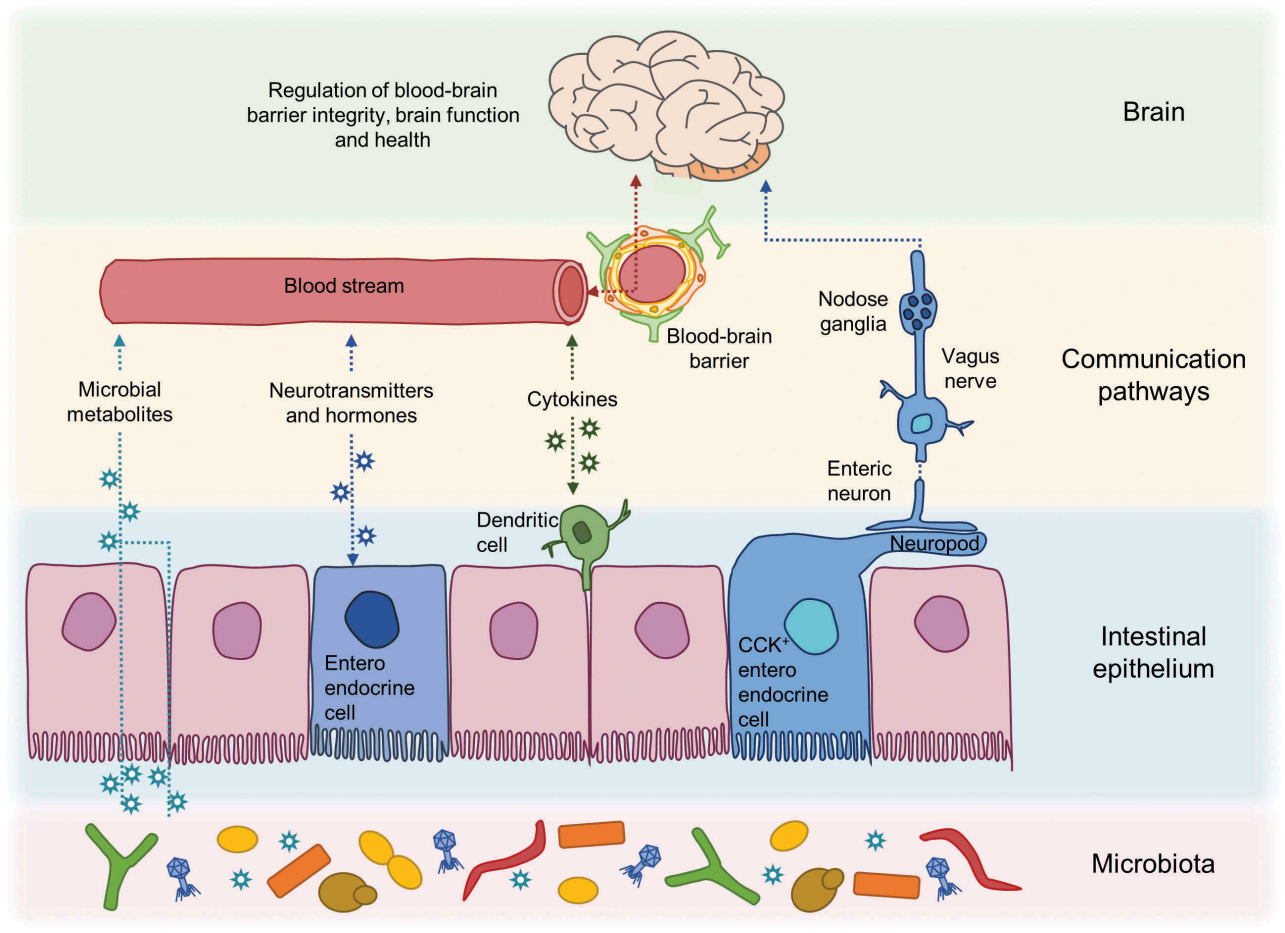
Pathways of communication along the gut-microbiota-brain axis. A complex interplay of epithelial, immune and neural cell signalling networks is involved in sensing and communicating changes in microbial metabolites in the gut and the brain involving both circulatory and neural routes.

Intestinal metabolic health status is relayed to the brain by both neural and circulatory routes (Figure 2). Bidirectional gut-brain neural relays control satiety signaling and appetite regulation.^94^ Detection of luminal metabolites by intestinal enteroendocrine cells is communicated to the brain via activation of the vagus nerve,^95^ by epithelial cell-derived hormones and neurotransmitters,^96,97^ and via neural routes, including neuroepithelial connections.^98,99^ Dietary metabolites and epithelial-derived soluble factors may also travel via the bloodstream to the BBB, where a variety of nutrient transporters and specific receptors expressed on BMECs^100,101^ facilitate the translocation of specific nutrients (e.g. glucose, lactate) across the barrier into the brain parenchyma, and allow for binding of circulating peptide hormones and other factors which can modulate brain activity. The microbial metabolites discussed above including SCFAs, TMAs, amino acid metabolites, and vitamins can travel via or modulate these pathways to affect enteroendocrine, neuronal, and immune cells in the gut, liver, kidneys, pancreas, and brain.

### Endocrine and neuroendocrine signaling

In the intestinal epithelium, specialized sensory enteroendocrine cells express a range of G-protein-coupled receptors (GPCR), transporters, and other receptors for fatty acids, carbohydrates, peptides, amino acids, and phytochemicals. Binding of bacterial products to these receptors induces the release of peptide hormones (including the incretin hormones GLP-1 and GIP, and neuropeptides CCK, PYY, and serotonin) which can act locally on enteric neurons, or enter the circulation and activate neurons innervating the portal vein, to signal to the brainstem via the vagus nerve. It is possible that some circulating hormones could reach and act directly on the brain. GLP-1 for example may be able to reach the brain either by crossing the blood-brain barrier^102^ or by acting at circumventricular structures.^103^ However, the short half-life of peptide hormones in the circulation,^104^ the excitation of enteric neurons and the vagus in response to metabolites and gut hormones,^105,106^ and the speed of responses to nutrients,^107^ reinforce the importance of neural signaling networks in communicating signals from the gut to the brainstem.^108^ Enteric neurons express receptors for SCFAs^109^ and bile acids^110^ facilitating metabolite regulation of gastrointestinal motility^111^ and allowing fast relay of information to the brain about metabolites crossing the epithelial barrier, and the concentrations of those metabolites in the gut lamina propria. For example, administration of luminal glucose activates both enteroendocrine cells and neural cells of the myenteric plexus, nodose ganglion and brainstem.^112^

Recent studies reveal direct physical associations between the epithelium and enteric nerves. Some enteroendocrine cells exhibit ‘neuropod’ processes which directly synapse with local neural networks, forming ‘neuroepithelial units’ which can and transmit chemical and electrical signals in response to metabolite binding.^99,113^ At present, the relative balance and importance of the circulatory, neural, and neuro-epithelial routes in relaying luminal information to the brain are unknown. Given the complexity within the gastrointestinal tract luminal environment, the summation and combination of all routes may be required to accurately convey varying spatiotemporal conditions and fluctuating microbial and metabolic states.

Encouragingly, the ability to map connectivity between neurons manipulate neural activity in real time, and measure neural activity during behavior has produced a wealth of data approaching a “wiring diagram” of the bidirectional gut-brain control of appetite, able to describe and predict the effects of dietary nutrients, common hormones and regulators.^94^ Building on this, recent studies are attempting to connect altered bacterial metabolism to specific neural signaling affecting brain functions. For example, Schretter and colleagues^114^ describe how locomotor hyperactivity in germfree Drosophila (manifested as increased walking speed and daily activity) can be rescued by altering sugar metabolism, either by colonization with *L. brevis* or by administration of *L. brevis*-derived xylose isomerase. The behavioral hyperactivity effect in germfree flies was shown to be mediated by octapaminergic neurons, as specific stimulation of these neurons or exogenous administration of octopamine (the invertebrate counterpart of noradrenaline) prevented the rescue by xylose isomerase.^114^ In mouse models, *in vivo* cell-connectivity tracing and enteroendocrine-neural co-cultures have shown that glucose stimulation of CCK-positive enteroendocrine cells activates vagal nodose neurons by glutamate-based neurotransmission.^99^ What impact this signaling might have on regulation of BBB integrity and neurodegenerative disease remains to be determined, but it is an exciting step forward in connecting specific gut epithelial-neural circuits and developing the necessary understanding to trace microbe-to-brain communication.

### Immune and neurotransmitter signaling

In addition to their actions on enteroendocrine and neural cells, metabolites can act via GPCR and other receptors to direct the development and normal functioning of the mucosal immune system; regulating the recruitment, development, maintenance, and activation of immune cells. The balance of dietary and bacterial metabolites is important for maintaining immune tolerance, and in directing inflammatory responses in the gut.

There are three main routes by which metabolite-immune cell interactions in the gut may be relayed to the brain. The first is via the secretion of cytokines and other immune signaling molecules into the bloodstream which ultimately reach the BBB. Breakdown of the epithelial barrier, resulting from dysbiosis, infection, damage, or age-associated degeneration allows ingress of bacteria, their metabolites, and other components such as toxins and lipopolysaccharides (LPS), triggering inflammatory immune responses and enteric neuronal damage.^115^ If not resolved, continuous or remitting intestinal inflammation (a hallmark of inflammatory bowel disease) can promote a state of chronic systemic inflammation characterized by elevated serum TNF, IL-6, and IL-1β, disrupting the BBB by increasing the permeability of the BMEC layer^116^ and allowing solutes and toxins into the brain. Increased systemic and brain inflammation also promotes immune cell extravasation^117^ and stimulates inflammatory signalling by cells of the NVU and brain microglia,^118^ perpetuating a vicious cycle of brain inflammation and neurodegeneration. *In vivo* infection models show that elevated TNF drives brain pathology in a Drosophila model of enterobacteria infection-aggravated AD.^119^ In mice, blocking TNF uptake at the BBB by inhibiting TNF receptor 1 (TNFR1) can reduce immune cell infiltration and ameliorate disease severity in a model of MS.^120^ Metabolite-immune cell interactions, intestinal barrier dysfunction, and bacterial infection can therefore be important early events in initiating neurodegenerative disease by altering gut-to-brain cytokine signaling and immune cell trafficking.

In the second route, metabolite induced secretion of immune cell cytokines and neurotransmitters may act locally on enteric neurons innervating the gut and alter vagus nerve signaling to the brain. With advancing age, a primary risk factor for neurodegenerative diseases, a shift in the polarisation of intestinal macrophages and their cytokine production alters enteric neural responses to inflammatory signals and increases apoptosis and loss of enteric neurons.^121^ Loss of enteric neurons not only alters intestinal functions but has implications for the ability of the gut to communicate with the brain. Furthermore, the cytokine milieu affects the functioning of enteric glial cells that support and regulate enteric neuronal functions by production of glial cell-derived neurotrophic factors (GDNFs). Glial cell GDNF production in response to microbial-immune cell signaling,^122^ and potentially via SCFA-GPCR activation,^123,124^ induces innate lymphoid type 3 (ILC3) cells to produce IL-22, promoting protection and restoration of the epithelial barrier.^125^ These cells are therefore important in integrating microbial and host signalling in neuro-immune responses in the intestinal mucosa.

Along with immunoregulatory cytokines, binding of bacterial metabolites to intestinal cells can also trigger the release of local peptide neurotransmitters, including PYY and serotonin, for which enteric neurons express specific receptors.^126,127^ For example, decarboxylation of tryptophan to tryptamine by *Clostridium sporogenes* induces enterochromaffin cells to release serotonin, which acts on enteric neurons to stimulate gastrointestinal motility.^128,129^Serotonin is also involved in regulating neurogenesis during development^130^ but detangling its role in adult neurodegeneration is complicated by the multitude of serotonin receptor subtypes expressed in the mammalian brain.^131^ Gut microbes may also trigger neurotransmitter release via Toll-Like Receptor (TLR) signaling on epithelial, immune and neuronal cells.^132,133^ Finally, bacterial metabolites may act directly as neurotransmitters, acting locally and in the brain. *Lactobacillus* and *Bifidobacterium* can metabolize glutamate to produce GABA, a major inhibitory neurotransmitter of the brain^68,134^ and modulator of gut functions.^135^ Various enteric bacteria are also able to synthesize and release nitric oxide,^136^ acetylcholine,^137^ noradrenaline,^138^ and dopamine.^139^ The emerging field of “psychobiotics”^140,141^ proposes to manipulate these neurotransmitter-producing bacteria to regulate gut and brain function.

In the third route, metabolite-activated immune cells may travel via the bloodstream to release soluble factors at the BBB which impacts its integrity and alters the inflammatory status of brain cells. Systemic inflammation and elevated circulating TNF promote extravasation of leukocytes and macrophages to the brain by upregulating adhesion molecules, chemokines, and matrix metalloproteases at the BBB and in the brain,^117,142^ and by downregulating tight junctions. At steady state, during infection, and following brain injury, T cells from the gut can traffic to the brain.^143,144^ Circulating immune cells may cross the BBB and choroid plexus to enter the brain and local draining lymph nodes^145,146^ or infiltrate via meningeal vascular channels.^147^ SCFA signaling may mitigate some of these effects, as altered lymphocyte migration from the gut in response to SCFAs can ameliorate uveitis^148^ and modulation of the microbiota and its metabolites can impact neuroinflammation, and neurodegeneration.

### Microbiota-gut-brain pathways in neurodegenerative disease

Multiple neurological and neurodegenerative disease are now being associated with gut dysbiosis, following infection, inflammation or antibiotic use, although the mechanisms of gut-brain transmission of pathology have so far remained elusive. PD is of particular interest in this regard as a proposed etiology has developed from a growing collection of animal and epidemiological studies which link PD with pathogenic alpha synuclein (αSyn) protein production and accumulation in the gastrointestinal tract. A proposed sequence of events involves age- infection-, or dysbiosis-driven αSyn accumulation in the intestine, leading to trans-synaptic transmission of pathological forms of αSyn from enteric neurons to the vagus nerve, and retrograde axonal transport along the vagus to the brainstem.^149-151^ This model draws on autopsy studies of Lewy bodies, and observations that onset of gastrointestinal symptoms in PD patients, and detection of pathological α-synuclein (αSyn) in gastrointestinal tissues in animal models of PD, are seen prior to the onset of motor symptoms. Also, rodent studies in which αSyn expression was chemically induced in enteric neurons, or in which recombinant αSyn was delivered to the gastric and/or duodenal wall, report subsequent αSyn detection in the vagal nerve and multiple brain regions.^152-155^ The identification of αSyn also within intestinal enteroendocrine cells^156^ is interesting in light of the recently described synaptic connections between subsets of these cells and vagal afferents,^113^ providing a gut lumen-to brain communication pathway.

What events may trigger the initial generation of pathogenic αSyn in the gut, and whether αSyn propagation from the intestine to the vagus and/or brainstem occurs in humans and is causative in human PD pathology remain unknown. While transmission of αSyn via the vagal route has received the most attention, gut-brain transmission by other neural pathways (e.g. the splanchnic nerve innervating the colon, olfactory neurons^157^) or by trafficking immune cells is also possible. Moreover, vagal transmission is not absolutely required for PD development, as vagotomy delays but does not prevent PD-like pathology in mice^153^ and while some human epidemiological studies suggest vagotomy is associated with lower risk of developing PD,^158^ others do not.^159,160^ The human appendix, which houses a diverse microbiome,^161^ and in which pathology-associated αSyn can be also detected, has also been suggested as a site of gastrointestinal αSyn seeding.^162^ While the history of appendectomy was associated with delayed onset of PD symptoms in a small late-onset cohort^163^ a larger study found no effect,^164^ and others find either no effect or a slightly increased risk of developing PD 5–10 years post-appendectomy.^165,166^ As a reservoir of microbes and immune cells, the appendix could still have a role in modulating PD risk by influencing microbial and immune homeostasis. Given the interaction between gut microbes, enteroendocrine cells, enteric neurons and the enteric immune system discussed above, and recent evidence that bacterial proteins can enhance αSyn aggregation in animal models,^167^ the activities of specific gut microbes or a more general ‘microbial dysbiosis’ could conceivably play a role in the transition from benign to pathogenic αSyn states.

In summary, a complex interplay of signalling between epithelial, immune, and neural cell signalling networks is involved in translating microbial metabolite changes in the gut and the brain, and may be involved in the etiology of neurodegenerative disease. Progress is being made in trying to dissect these interactions, but a better understanding of microbe-brain communication now requires cross-disciplinary multi-organ-based investigations.

## Modulation of the gut microbiota as a potential therapeutic tool for promotion and restoration of brain health

Disruption of the complex ecosystem of the intestinal microbiota is now implicated in multiple conditions and diseases of both the intestine and the brain, and some of the routes by which microbes may act on the gut-brain axis are beginning to be unravelled. Manipulating or shaping the microbiota is therefore attracting attention as a viable strategy to prevent or treat various extra-intestinal diseases. The beneficial effects of an ‘optimized’ gut microbiota would include immune and epithelial homeostasis, enteric nervous system regulation, and optimal digestion and metabolism. This would theoretically protect against neurodegenerative disease by preventing metabolic, cardiovascular and inflammatory conditions which are associated with neurodegeneration but, as discussed above, could also protect BBB integrity and brain function by more direct means. Current therapeutic approaches under consideration include modifying the existing microbial composition in the gut by: altering nutrient availability to promote the growth of particular classes or species of bacteria (prebiotics); introducing or expanding ‘beneficial’ species (probiotics); or by wholesale transplant of entire communities or portions of communities from other intestinal donors (faecal microbial transplantation and more selective stool transplants). Below we consider how some of these approaches are beginning to be tested, and the potential for their use in improving blood-brain-barrier homeostasis and brain health.

### Promoting growth of beneficial microbes via prebiotics

Prebiotics are non-digestible short-chain carbohydrates that, when ingested selectively, stimulate the development of some health-promoting colonic bacteria.^168^ The nutritional properties exhibited by prebiotics derive from their resistance to hydrolysis in the upper gastrointestinal tract and their fermentation in the large intestine by mainly anaerobic bacteria.^169^ Common prebiotics include disaccharides (lactulose), oligosaccharides (fructooligosaccharides, FOS, galactooligosaccharides, GOS) and polysaccharides (inulin).^170^ Other compounds that can also be considered as prebiotics are resistant starches, pectin, whole grains, and polyphenols.^171^ Once in the gut, prebiotic compounds are metabolized by the host microbiota and resulting metabolites, such as SCFAs, act on the brain through different pathways contributing to an effect on BBB permeability and neurological health (described above). Several animal studies have analyzed the effect of prebiotics on brain function^172-176^ including modulating mood and stress responses by activating or supressing the hypothalamic-pituitary-adrenal (HPA) or “stress” axis. Serum and salivary cortisol are reliable indicators of HPA-activation (stress) and can be modulated by pre- and/or probiotics. The raised levels of circulating cortisol in germfree rodents^177^ are reduced following the administration of probiotics.^178^ In healthy mice, supplementation with long-chain FOS or short-chain GOS, which promote the growth of beneficial species such as Bifidobacteria,^172^ decreased anxiety-related behaviors and increased social behavior.^179^ Few studies investigating the benefit of prebiotics on human brain function have been published to date, and are so far inconclusive. One example in healthy adult volunteers described reduced stress response (salivary cortisol awakening response) after GOS intake.^180^ In preterm infants, supplementation with different prebiotics showed improvement in brain development when compared to a control group.^181^ Synergistic combinations of prebiotics and probiotics (synbiotics) have been found to improve age-related memory impairment in rats^182^ and are now being trialled in human clinical studies to promote intestinal health.^183,184^ One simple goal arising from such studies is devising optimized healthy diets which include all the required nutrients and prebiotics needed to decrease the number of harmful gut microbiota, and to increase the concentration of microorganisms producing metabolites which have beneficial effects on immunologic, metabolic and brain function to maximize health.

### Changing the gut microbiota composition via probiotics

Probiotics are non-pathogenic living microorganisms added to, or naturally occurring in, food products, that confer a health benefit to the host when administered in adequate amounts.^185^ Examples include gut include species of the genera *Lactobacillus, Bifidobacterium, Lactococcus, Streptococcus*, and *Enterococcus*.^186^ There is increasing interest in exploring the beneficial features of next-generation probiotics or live therapeutic products which include species not traditionally considered as probiotics, such as *Akkermansia muciniphila*,^187^ *Faecalibacterium prausnitzii*,^188^ species of the genus *Bacteroides*,^189^ and genetically modified *Lactococcus* strains carrying beneficial traits.^190^ Recent evidence suggests that probiotic bacterial viability is not necessary to develop health-promoting effects in the host.^191^ The administration of inactivated probiotic microorganisms, known as paraprobiotics, has been shown to confer benefits to the host without the risk of enhanced inflammatory responses in immunocompromised individuals.^192,193^ In line with this concept, the term postbiotics, also known as metabiotics or simply metabolites, emerged to refer to bioactive soluble factors secreted by live bacteria or released after bacterial lysis, reinforcing the idea that cell viability is not essential to induce healthy effects.^191^

Probiotic treatments have been associated with modulating mood and anxiety in animal models and humans. The first reported trial of using probiotic bacteria to treat mental health conditions was published in the early 20^th^ century and described the use of lactic acid bacteria to successfully treat melancholia and constipation.^194^ The term “psychobiotics” has since been coined to describe bacteria which, when ingested in adequate amounts, have a positive mental health effect.^140^ Several human interventions provided evidence that psychobiotics can alter mental state. The intake of *Lactobacillus helveticus* and *Bifidobacterium longum* reduced 24-hour urinary free cortisol, a biomarker for stress response, in healthy volunteers.^195^ Healthy students consuming fermented milk containing *Lactobacillus casei* strain Shirota had lower plasma cortisol compared to placebo group on the day before an examination,^196^ and a probiotic strain of *Lactobacillus rhamnosus* exhibited a protective effect on symptoms of postpartum depression.^197^ One possible mechanism for the mood-altering effects of psychobiotics is by enhancing production of neurotransmitters, such as GABA and glutamate which control neural excitation-inhibition balance, and BDNF which is implicated in learning processes and control of fear.^198^ Another possible mechanism is by altering the balance of circulating pro-and anti-inflammatory cytokines, restoring inflammation-induced BBB permeability, preventing potentially harmful material from entering the brain,^199^ and promoting mental health and psychological resilience.^140^

Probiotic modulation has also shown promise in alleviating symptoms of PD. Delivery of some probiotic cultures has shown beneficial effects on gastrointestinal symptoms in PD patients,^200,201^ while one study delivering a *Bifidobacteria/Lactobacilli* mix found improvements in motor scores.^202^ Probiotics may also prove useful in treating AD. Delivery of *Bifidobacteria/Lactobacilli* mixtures has shown several benefits in mouse and rat models of AD, including improvements in memory and learning tests, reduced measures of oxidative stress, reduction in histopathological markers in the brain,^203,204^and restoration of neuronal cell proteolytic functions.^205^ Some small-scale exploratory human studies comparing AD patients before and after probiotic supplementation, again using *Bifidobacteria/Lactobacilli* mixtures, report improved mini-mental state examination (MMSE) scores and improvement in some metabolic and inflammatory markers.^206,207^ Two recent randomized, double-blinded, placebo-controlled clinical trials show improved MMSE cores, reduced serum C-reactive protein (an inflammatory marker) and improved insulin metabolism in patients receiving probiotic mixture containing *Bifidobacteria* and *Lactobacilli* species.^208,209^ As with PD, the mechanisms of probiotic-promoted neuroprotective effects in AD studies remain to be determined but may reflect positive effects on gut barrier health leading to improved immune and metabolic health (e.g. protection against type-II diabetes, decreased systemic inflammation), which in turn protects against BBB breakdown and neural pathology.

### Changing the gut microbiota via fecal microbiota transplantation (FMT)

FMT is the therapeutic administration of fecal material containing gut microbiota from a healthy donor to a recipient with a dysbiosis-related condition, via enema, nasogastric, nasoenteric or endoscopic routes, to restore normal diversity and function in the microbial community.^210^ FMT was first described in a medical manual for emergencies written by the Chinese alchemist Ge Hong in the fourth century, who reported that people suffering from food poisoning and diarrhea recovered after oral administration of fecal suspensions (‘yellow dragon soup’).^211^ A more recent and well-documented use of FMT is the treatment of patients with *Clostridium difficile* infection, with efficacy rates of approximately 90%.^212^ FMT has also been used to treat ulcerative colitis but with an effectiveness of lower than 25%.^213^ The overall results of several randomized controlled trials using FMT to treat irritable bowel syndrome are so far inconclusive regarding efficacy in improving severity scores.^214^ Due to the bidirectional signalling interactions along the microbiota-gut-brain axis, FMT has also been suggested as a potential treatment for extra-intestinal disorders, including neurological conditions. Bercik and colleagues^215^ published one of the first studies using FMT to modulate brain function and modulate host behavior in mice. They colonized germfree BALB/c mice, usually a timid strain, with microbiota from the more exploratory NIH Swiss strain. The BALB/c mice displayed less timid and more exploratory behaviour after FMT, associated with higher levels of BDNF in the brain. In the reverse experiment, colonization of germfree NIH Swiss mice with BALB/c microbiota reduced exploratory behavior, suggesting the gut microbiota play a key role in behavior.^215^ In support of this study, Zheng and colleagues^216^ showed that germfree mice transplanted with microbiota from major depressive disorder patients displayed depression-like behavior. Kelly and colleagues^217^ also found physiological and behavioral features of depression in microbiota-depleted rats after transplantation with fecal microbiota from depressed patients, as well as a dysregulation in tryptophan metabolism, similar to that reported in autism, schizophrenia and neurodegenerative diseases.^218,219^ In a disease model, FMT from normal mice has neuroprotective effects in a mouse model of PD, including reducing TNF-driven neuroinflammation and increasing brain levels of dopamine and serotonin, leading to improvement in motor symptoms.^220^ Currently, studies focusing on the effect of FMT on the neurodegenerative disease in humans are lacking. Anecdotally, neurologic improvement in one patient with PD has been reported following FMT for chronic constipation.^221^ Given the possible gut-brain propagation of pathogenic αSyn discussed above, the use of FMT to modulate the intestinal production of αSyn could be protective against PD by providing a ‘protective’ microbiota, which either does not promote aberrant protein folding or outcompetes pathogenic strains, or by maintaining immune, neural and epithelial barrier homeostasis such that any aberrant protein folding in the gut lumen is contained locally. However, safety remains a major concern in translating FMT research to humans. Stool donors and samples for FMT are currently tested for different potentially pathogenic bacteria, viruses, parasites, and other microorganisms,^222^ but this is far from knowing the complete microbial composition of the sample to be transplanted, raising both safety concerns and issues over how to interpret results. The delivery of selective microbial mixtures, as in stool substitute transplant therapy (SSTT) or microbial ecosystem therapeutics (MET), in which purified beneficial intestinal bacterial cultures derived from a single healthy human donor are delivered into a patient’s gastrointestinal tract, could minimize the potential risk of pathogenicity, not only due to infection but also to metabolic disease development or oncogenic potential. By inducing large-scale shifts in the intestinal microbiota by methods such as FMT and SSTT, metabolite production in the gut and subsequent availability in circulation may also be significantly altered. One recent study of African turquoise killifish (*Nothobranchius furzeri*) using metabolomic analysis of intestine, brain, liver, heart, skeletal muscle, serum, and stool, found important metabolic pathways are modified during killifish aging and that these changes could in part be reversed by gut microbiota transfers from young donors.^223^ New work on metabolomics will be vital to understanding the complex metabolic interactions between host and microbes and how metabolic profiles are associated with health and disease. By harnessing such methods, and combining this information with microbial ‘omics and systems biology approaches, it may be possible to identify beneficial vs detrimental microbial and metabolic profiles and pathways for gut and brain health, and to work towards optimized personal therapies to achieve beneficial conditions using a combination of microbial and dietary intervention. These and other improvements in our understanding of microbe–host interactions at the gut and brain barriers will hopefully accelerate research in the use of microbiota-modulating therapies to prevent and treat neurological and neurodegenerative disease.

## Conclusions

Gut microbiome research is rapidly expanding, with recent advances in high-throughput ‘omics technologies, providing us with a better understanding of the composition and functionality of such a complex ecosystem. Further investigation of bacterial metabolites and their effects on hormone production, immune signalling, and neural function will enable a fuller understanding of brain responses to age- and disease-associated alterations in the microbiota. Despite our current limited knowledge of specific mechanisms, dietary and microbial modulation are showing promise as potential strategies to tackle some neurodegenerative and neurological diseases. A deeper understanding of gut microbial ecology, metabolism, and signalling networks within the host may lead to a new generation of microbiome-targeted strategies, both for disease treatment and prevention.

## References

[1] Duda-Chodak A, Tarko T, Satora P, Sroka P. Interaction of dietary compounds, especially polyphenols, with the intestinal microbiota: a review. Eur J Nutr. 2015;54:325–341. doi:10.1007/s00394-015-0852-y. 25672526 PMC4365176

[2] Sender R, Fuchs S, Milo R. Revised estimates for the number of human and bacteria cells in the body. PLoS Biol. 2016;14:e1002533. doi:10.1371/journal.pbio.1002533. 27541692 PMC4991899

[3] Laval L, Martin R, Natividad JN, Chain F, Miquel S, Desclee de Maredsous C, Capronnier S, Sokol H, Verdu EF, van Hylckama Vlieg JET, et al. *Lactobacillus rhamnosus* CNCM I-3690 and the commensal bacterium *Faecalibacterium prausnitzii* A2-165 exhibit similar protective effects to induced barrier hyper-permeability in mice. Gut Microbes. 2015;6:1–9. doi:10.4161/19490976.2014.990784. 25517879 PMC4615674

[4] Nash V, Ranadheera CS, Georgousopoulou EN, Mellor D, Panagiotakos DB, McKune A, Kellett J, Naumovski N. The effects of grape and red wine polyphenols on gut microbiota – a systematic review. Food Res Int (Ottawa, Ont). 2018;113:277–287. doi:10.1016/j.foodres.2018.07.019. 30195522

[5] Fung TC, Olson CA, Hsiao EY. Interactions between the microbiota, immune and nervous systems in health and disease. Nat Neurosci. 2017;20:145–155. doi:10.1038/nn.4476. 28092661 PMC6960010

[6] Braniste V, Al-Asmakh M, Kowal C, Anuar F, Abbaspour A, Toth M, Korecka A, Bakocevic N, Ng LG, Kundu P, et al. The gut microbiota influences blood-brain barrier permeability in mice. Sci Transl Med. 2014;6:263ra158. 10.1126/scitranslmed.3009759 PMC4396848 25411471

[7] Fröhlich EE, Farzi A, Mayerhofer R, Reichmann F, Jacan A, Wagner B, Zinser E, Bordag N, Magnes C, Fröhlich E, et al. Cognitive impairment by antibiotic-induced gut dysbiosis: analysis of gut microbiota-brain communication. Brain Behav Immun. 2016;56:140–155. doi:10.1016/j.bbi.2016.02.020. 26923630 PMC5014122

[8] Spielman LJ, Klegeris A. The role of insulin and incretins in neuroinflammation and neurodegeneration. Immunoendocrinology. 2014;1:e391. doi:10.14800/immunoendocrinology.391.

[9] Block ML, Hong JS. Microglia and inflammation-mediated neurodegeneration: multiple triggers with a common mechanism. Prog Neurobiol. 2005;76:77–98. doi:10.1016/j.pneurobio.2005.06.004. 16081203

[10] Block ML, Zecca L, Hong JS. Microglia-mediated neurotoxicity: uncovering the molecular mechanisms. Nat Rev Neurosci. 2007;8:57–69. doi:10.1038/nrn2038. 17180163

[11] Campos-Bedolla P, Walter FR, Veszelka S, Deli MA. Role of the blood-brain barrier in the nutrition of the central nervous system. Arch Med Res. 2014;45:610–638. doi:10.1016/j.arcmed.2014.11.018. 25481827

[12] Harach T, Marungruang N, Dutilleul N, Cheatham V, Mc Coy K, Neher J, Jucker M, Fåk F, Bolmont T. Reduction of Alzheimer’s disease beta-amyloid pathology in the absence of gut microbiota. arXiv:1509.02273v2. 2015.

[13] Minter MR, Zhang C, Leone V, Ringus DL, Zhang X, Oyler-Castrillo P, Musch MW, Liao F, Ward JF, Holtzman DM, et al. Antibiotic-induced perturbations in gut microbial diversity influences neuro-inflammation and amyloidosis in a murine model of Alzheimer’s disease. Sci Rep. 2016;6:30028. doi:10.1038/srep30028. 27443609 PMC4956742

[14] Scheperjans F, Aho V, Pereira PA, Koskinen K, Paulin L, Pekkonen E, Haapaniemi E, Kaakkola S, Eerola-Rautio J, Pohja M, et al. Gut microbiota are related to Parkinson’s disease and clinical phenotype. Mov Disord. 2015;30:350–358. doi:10.1002/mds.26069. 25476529

[15] Sampson TR, Debelius JW, Thron T, Janssen S, Shastri GG, Ilhan ZE, Challis C, Schretter CE, Rocha S, Gradinaru V, et al. Gut microbiota regulate motor deficits and Neuroinflammation in a model of Parkinson’s disease. Cell. 2016;167:1469–80 e12. doi:10.1016/j.cell.2016.11.018. 27912057 PMC5718049

[16] Stern L, Gautier R. Recherches Sur Le Liquide Céphalo-Rachidien: I.–les Rapports Entre Le Liquide Céphalo-Rachidien et la Circulation Sanguine. Arch Int Physiol. 1921;17:138–192. doi:10.3109/13813452109146211.

[17] Brightman MW, Reese TS. Junctions between intimately apposed cell membranes in the vertebrate brain. J Cell Biol. 1969;40:648–677. doi:10.1083/jcb.40.3.648. 5765759 PMC2107650

[18] Reese TS, Karnovsky MJ. Fine structural localization of a blood-brain barrier to exogenous peroxidase. J Cell Biol. 1967;34:207–217. doi:10.1083/jcb.34.1.207. 6033532 PMC2107213

[19] Ruck T, Bittner S, Meuth SG. Blood-brain barrier modeling: challenges and perspectives. Neural Regen Res. 2015;10:889–891. doi:10.4103/1673-5374.158342. 26199600 PMC4498345

[20] Hawkins BT, Davis TP. The blood-brain barrier/neurovascular unit in health and disease. Pharmacol Rev. 2005;57:173–185. doi:10.1124/pr.57.2.4. 15914466

[21] Ballabh P, Braun A, Nedergaard M. The blood-brain barrier: an overview: structure, regulation, and clinical implications. Neurobiol Dis. 2004;16:1–13. doi:10.1016/j.nbd.2003.12.016. 15207256

[22] Vorbrodt AW, Dobrogowska DH. Molecular anatomy of intercellular junctions in brain endothelial and epithelial barriers: electron microscopist’s view. Brain Res Brain Res Rev. 2003;42:221–242. 12791441 10.1016/s0165-0173(03)00177-2

[23] Rustenhoven J, Jansson D, Smyth LC, Dragunow M. Brain pericytes as mediators of neuroinflammation. Trends Pharmacol Sci. 2017;38:291–304. doi:10.1016/j.tips.2016.12.001. 28017362

[24] Moura RP, Almeida A, Sarmento B. The role of non-endothelial cells on the penetration of nanoparticles through the blood brain barrier. Prog Neurobiol. 2017;159:39–49. doi:10.1016/j.pneurobio.2017.09.001. 28899762

[25] Abbott NJ, Ronnback L, Hansson E. Astrocyte-endothelial interactions at the blood-brain barrier. Nat Rev Neurosci. 2006;7:41–53. doi:10.1038/nrn1824. 16371949

[26] Liebner S, Dijkhuizen RM, Reiss Y, Plate KH, Agalliu D, Constantin G. Functional morphology of the blood-brain barrier in health and disease. Acta Neuropathol. 2018;135:311–336. doi:10.1007/s00401-018-1815-1. 29411111 PMC6781630

[27] Zhang J, Ke KF, Liu Z, Qiu YH, Peng YP. Th17 cell-mediated neuroinflammation is involved in neurodegeneration of abeta1-42-induced Alzheimer’s disease model rats. PLoS One. 2013;8:e75786. doi:10.1371/journal.pone.0075786. 24124514 PMC3790825

[28] Sommer A, Maxreiter F, Krach F, Fadler T, Grosch J, Maroni M, Graef D, Eberhardt E, Riemenschneider MJ, Yeo GW, et al. Th17 lymphocytes induce neuronal cell death in a human iPSC-based model of Parkinson’s disease. Cell Stem Cell. 2018;23:123–31 e6. doi:10.1016/j.stem.2018.06.015. 29979986

[29] Sagare AP, Bell RD, Zhao Z, Ma Q, Winkler EA, Ramanathan A, Zlokovic BV. Pericyte loss influences Alzheimer-like neurodegeneration in mice. Nat Commun. 2013;4:2932. doi:10.1038/ncomms3932. 24336108 PMC3945879

[30] Li H, Sun J, Du J, Wang F, Fang R, Yu C, Xiong J, Chen W, Lu Z, Liu J. *Clostridium butyricum* exerts a neuroprotective effect in a mouse model of traumatic brain injury via the gut-brain axis. Neurogastroenterol Motil. 2018;30:e13260. doi:10.1111/nmo.2018.30.issue-5. 29193450

[31] Li H, Sun J, Wang F, Ding G, Chen W, Fang R, Yao Y, Pang M, Lu Z-Q, Liu J. Sodium butyrate exerts neuroprotective effects by restoring the blood-brain barrier in traumatic brain injury mice. Brain Res. 2016;1642:70–78. doi:10.1016/j.brainres.2016.03.031. 27017959

[32] Quinn M, McMillin M, Galindo C, Frampton G, Pae HY, DeMorrow S. Bile acids permeabilize the blood brain barrier after bile duct ligation in rats via Rac1-dependent mechanisms. Dig Liver Dis. 2014;46:527–534. doi:10.1016/j.dld.2014.01.159. 24629820 PMC4065628

[33] Hoyles L, Snelling T, Umlai UK, Nicholson JK, Carding SR, Glen RC, McArthur S. Microbiome-host systems interactions: protective effects of propionate upon the blood-brain barrier. Microbiome. 2018;6:55. doi:10.1186/s40168-018-0432-5. 29562936 PMC5863458

[34] Ganapathy V, Thangaraju M, Prasad PD, Martin PM, Singh N. Transporters and receptors for short-chain fatty acids as the molecular link between colonic bacteria and the host. Curr Opin Pharmacol. 2013;13:869–874. doi:10.1016/j.coph.2013.08.006. 23978504

[35] Reichardt N, Duncan SH, Young P, Belenguer A, McWilliam Leitch C, Scott KP, Flint HJ, Louis P. Phylogenetic distribution of three pathways for propionate production within the human gut microbiota. Isme J. 2014;8:1323–1335. doi:10.1038/ismej.2014.14. 24553467 PMC4030238

[36] Salminen S, Bouley C, Boutron-Ruault MC, Cummings JH, Franck A, Gibson GR, Isolauri E, Moreau MC, Roberfroid M, Rowland I. Functional food science and gastrointestinal physiology and function. Br J Nutr. 1998;80:S147–71. 9849357 10.1079/bjn19980108

[37] Macfabe DF. Short-chain fatty acid fermentation products of the gut microbiome: implications in autism spectrum disorders. Microb Ecol Health Dis. 2012;23:19260. doi:10.3402/mehd.v23i0.19260. 10.3402/mehd.v23i0.19260 PMC3747729 23990817

[38] Kim HJ, Rowe M, Ren M, Hong JS, Chen PS, Chuang DM. Histone deacetylase inhibitors exhibit anti-inflammatory and neuroprotective effects in a rat permanent ischemic model of stroke: multiple mechanisms of action. J Pharmacol Exp Ther. 2007;321:892–901. doi:10.1124/jpet.107.120188. 17371805

[39] Matt SM, Allen JM, Lawson MA, Mailing LJ, Woods JA, Johnson RW. Butyrate and dietary soluble fiber improve neuroinflammation associated with aging in mice. Front Immunol. 2018;9:1832. doi:10.3389/fimmu.2018.01832. 30154787 PMC6102557

[40] Fischer A, Radulovic M, Schrick C, Sananbenesi F, Godovac-Zimmermann J, Radulovic J. Hippocampal Mek/Erk signaling mediates extinction of contextual freezing behavior. Neurobiol Learn Mem. 2007;87:149–158. doi:10.1016/j.nlm.2006.08.003. 16979915 PMC1839930

[41] MacFabe DF, Cain DP, Rodriguez-Capote K, Franklin AE, Hoffman JE, Boon F, Taylor AR, Kavaliers M, Ossenkopp K-P. Neurobiological effects of intraventricular propionic acid in rats: possible role of short chain fatty acids on the pathogenesis and characteristics of autism spectrum disorders. Behav Brain Res. 2007;176:149–169. doi:10.1016/j.bbr.2006.07.025. 16950524

[42] Erny D, Hrabe de Angelis AL, Jaitin D, Wieghofer P, Staszewski O, David E, Keren-Shaul H, Mahlakoiv T, Jakobshagen K, Buch T, et al. Host microbiota constantly control maturation and function of microglia in the CNS. Nat Neurosci. 2015;18:965–977. doi:10.1038/nn.4030. 26030851 PMC5528863

[43] Zilberter Y, Zilberter M. The vicious circle of hypometabolism in neurodegenerative diseases: ways and mechanisms of metabolic correction. J Neurosci Res. 2017;95:2217–2235. doi:10.1002/jnr.24064. 28463438

[44] Emery DC, Shoemark DK, Batstone TE, Waterfall CM, Coghill JA, Cerajewska TL, Davies M, West NX, Allen SJ. 16S rRNA next generation sequencing analysis shows bacteria in Alzheimer’s post-mortem brain. Front Aging Neurosci. 2017;9:195. doi:10.3389/fnagi.2017.00077. 28676754 PMC5476743

[45] Readhead B, Haure-Mirande JV, Funk CC, Richards MA, Shannon P, Haroutunian V, Sano M, Liang WS, Beckmann ND, Price ND, et al. Multiscale Analysis of independent Alzheimer’s Cohorts finds disruption of molecular, genetic, and clinical networks by human Herpesvirus. Neuron. 2018;99:64–82 e7. doi:10.1016/j.neuron.2018.05.023. 29937276 PMC6551233

[46] Pisa D, Alonso R, Fernandez-Fernandez AM, Rabano A, Carrasco L. Polymicrobial infections in brain tissue from Alzheimer’s disease patients. Sci Rep. 2017;7:5559. doi:10.1038/s41598-017-05903-y. 28717130 PMC5514053

[47] Pisa D, Alonso R, Rabano A, Rodal I, Carrasco L. Different brain regions are infected with fungi in Alzheimer’s disease. Sci Rep. 2015;5:15015. doi:10.1038/srep15015. 26468932 PMC4606562

[48] Pisa D, Alonso R, Juarranz A, Rabano A, Carrasco L. Direct visualization of fungal infection in brains from patients with Alzheimer’s disease. J Alzheimers Dis. 2015;43:613–624. doi:10.3233/JAD-141386. 25125470

[49] Bensasson D, Dicks J, Ludwig JM, Bond CJ, Elliston A, Roberts IN, James SA. Diverse Lineages of *Candida albicans* live on old Oaks. Genetics. 2019;211:277–288. doi:10.1534/genetics.118.301482. 30463870 PMC6325710

[50] Lachance M-A, Boekhout T, Scorzetti G, Fell JW, Kurtzman CP. Candida Berkhout (1923). In: Kurtzman CP, Fell JW, Boekhout T, editors. The yeasts. Elsevier; 2011. p. 987–1278. doi:10.1016/B978-0-444-52149-1.00090-2.

[51] Bennett BJ, de Aguiar Vallim TQ, Wang Z, Shih DM, Meng Y, Gregory J, Allayee H, Lee R, Graham M, Crooke R, et al. Trimethylamine-N-oxide, a metabolite associated with atherosclerosis, exhibits complex genetic and dietary regulation. Cell Metab. 2013;17:49–60. doi:10.1016/j.cmet.2012.12.011. 23312283 PMC3771112

[52] Romano KA, Vivas EI, Amador-Noguez D, Rey FE. Intestinal microbiota composition modulates choline bioavailability from diet and accumulation of the proatherogenic metabolite trimethylamine-N-oxide. MBio. 2015;6:e02481. doi:10.1128/mBio.02481-14. 25784704 PMC4453578

[53] Del Rio D, Zimetti F, Caffarra P, Tassotti M, Bernini F, Brighenti F, Zini A, Zanotti I. The gut microbial metabolite trimethylamine-N-oxide is present in human cerebrospinal fluid. Nutrients. 2017;9:1053. 10.3390/nu9101053 PMC5691670 28937600

[54] Bae S, Ulrich CM, Neuhouser ML, Malysheva O, Bailey LB, Xiao L, Brown EC, Cushing-Haugen KL, Zheng Y, Cheng T-YD, et al. Plasma choline metabolites and colorectal cancer risk in the women’s health initiative observational study. Cancer Res. 2014;74:7442–7452. doi:10.1158/0008-5472.CAN-14-1835. 25336191 PMC4268282

[55] Koeth RA, Wang Z, Levison BS, Buffa JA, Org E, Sheehy BT, Britt EB, Fu X, Wu Y, Li L, et al. Intestinal microbiota metabolism of L-carnitine, a nutrient in red meat, promotes atherosclerosis. Nat Med. 2013;19:576–585. doi:10.1038/nm.3145. 23563705 PMC3650111

[56] Brugere JF, Borrel G, Gaci N, Tottey W, O’Toole PW, Malpuech-Brugere C. Archaebiotics: proposed therapeutic use of Archaea to prevent trimethylaminuria and cardiovascular disease. Gut Microbes. 2014;5:5–10. doi:10.4161/gmic.26749. 24247281 PMC4049937

[57] Gaci N, Borrel G, Tottey W, O’Toole PW, Brugere JF. Archaea and the human gut: new beginning of an old story. World J Gastroenterol. 2014;20:16062–16078. doi:10.3748/wjg.v20.i43.16062. 25473158 PMC4239492

[58] Nkamga VD, Lotte R, Chirio D, Lonjon M, Roger PM, Drancourt M, Ruimy R. Methanobrevibacter oralis detected along with *Aggregatibacter actinomycetemcomitans* in a series of community-acquired brain abscesses. Clin Microbiol Infect. 2018;24:207–208. doi:10.1016/j.cmi.2017.08.021. 28882726

[59] Dumas ME, Rothwell AR, Hoyles L, Aranias T, Chilloux J, Calderari S, Noll EM, Péan N, Boulangé CL, Blancher C, et al. Microbial-Host Co-metabolites are prodromal markers predicting Phenotypic Heterogeneity in behavior, obesity, and impaired glucose tolerance. Cell Rep. 2017;20:136–148. doi:10.1016/j.celrep.2017.06.039. 28683308 PMC5507771

[60] Lupachyk S, Watcho P, Stavniichuk R, Shevalye H, Obrosova IG. Endoplasmic reticulum stress plays a key role in the pathogenesis of diabetic peripheral neuropathy. Diabetes. 2013;62:944–952. doi:10.2337/db12-0716. 23364451 PMC3581201

[61] Matsumoto T, Nagase Y, Hirose J, Tokuyama N, Yasui T, Kadono Y, Ueki K, Kadowaki T, Nakamura K, Tanaka S. Regulation of bone resorption and sealing zone formation in osteoclasts occurs through protein kinase B-mediated microtubule stabilization. J Bone Miner Res. 2013;28:1191–1202. doi:10.1002/jbmr.1844. 23239117

[62] Tseng HC, Graves DJ. Natural methylamine osmolytes, trimethylamine N-oxide and betaine, increase tau-induced polymerization of microtubules. Biochem Biophys Res Commun. 1998;250:726–730. doi:10.1006/bbrc.1998.9382. 9784413

[63] Drewes G, Ebneth A, Preuss U, Mandelkow EM, Mandelkow E. MARK, a novel family of protein kinases that phosphorylate microtubule-associated proteins and trigger microtubule disruption. Cell. 1997;89:297–308. 9108484 10.1016/s0092-8674(00)80208-1

[64] Xu R, Wang Q. Towards understanding brain-gut-microbiome connections in Alzheimer’s disease. BMC Syst Biol. 2016;10(Suppl 3):63. doi:10.1186/s12918-016-0304-1. 27585440 PMC5009560

[65] Yang DS, Yip CM, Huang TH, Chakrabartty A, Fraser PE. Manipulating the amyloid-beta aggregation pathway with chemical chaperones. J Biol Chem. 1999;274:32970–32974. doi:10.1074/jbc.274.46.32970. 10551864

[66] Bose S, Cho J. Targeting chaperones, heat shock factor-1, and unfolded protein response: promising therapeutic approaches for neurodegenerative disorders. Ageing Res Rev. 2017;35:155–175. doi:10.1016/j.arr.2016.09.004. 27702699

[67] Wang S, Cui W, Zeng M, Ren Y, Han S, Li J. The increased release of amino acid neurotransmitters of the primary somatosensory cortical area in rats contributes to remifentanil-induced hyperalgesia and its inhibition by lidocaine. J Pain Res. 2018;11:1521–1529. doi:10.2147/JPR.S168008. 30147356 PMC6097504

[68] Barrett E, Ross RP, O’Toole PW, Fitzgerald GF, Stanton C. gamma-Aminobutyric acid production by culturable bacteria from the human intestine. J Appl Microbiol. 2012;113:411–417. doi:10.1111/j.1365-2672.2012.05344.x. 22612585

[69] Agus A, Planchais J, Sokol H. Gut microbiota regulation of tryptophan metabolism in health and disease. Cell Host Microbe. 2018;23:716–724. doi:10.1016/j.chom.2018.05.003. 29902437

[70] Williams BB, Van Benschoten AH, Cimermancic P, Donia MS, Zimmermann M, Taketani M, Ishihara A, Kashyap PC, Fraser JS, Fischbach MA. Discovery and characterization of gut microbiota decarboxylases that can produce the neurotransmitter tryptamine. Cell Host Microbe. 2014;16:495–503. doi:10.1016/j.chom.2014.09.001. 25263219 PMC4260654

[71] Yano JM, Yu K, Donaldson GP, Shastri GG, Ann P, Ma L, Nagler CR, Ismagilov RF, Mazmanian SK, Hsiao EY. Indigenous bacteria from the gut microbiota regulate host serotonin biosynthesis. Cell. 2015;161:264–276. doi:10.1016/j.cell.2015.02.047. 25860609 PMC4393509

[72] Clarke G, Grenham S, Scully P, Fitzgerald P, Moloney RD, Shanahan F, Dinan TG, Cryan JF. The microbiome-gut-brain axis during early life regulates the hippocampal serotonergic system in a sex-dependent manner. Mol Psychiatry. 2013;18:666–673. doi:10.1038/mp.2012.77. 22688187

[73] Desbonnet L, Garrett L, Clarke G, Bienenstock J, Dinan TG. The probiotic *Bifidobacteria infantis*: an assessment of potential antidepressant properties in the rat. J Psychiatr Res. 2008;43:164–174. doi:10.1016/j.jpsychires.2008.03.009. 18456279

[74] Rothhammer V, Mascanfroni ID, Bunse L, Takenaka MC, Kenison JE, Mayo L, Chao -C-C, Patel B, Yan R, Blain M, et al. Type I interferons and microbial metabolites of tryptophan modulate astrocyte activity and central nervous system inflammation via the aryl hydrocarbon receptor. Nat Med. 2016;22:586–597. doi:10.1038/nm.4106. 27158906 PMC4899206

[75] Alkhalaf LM, Ryan KS. Biosynthetic manipulation of tryptophan in bacteria: pathways and mechanisms. Chem Biol. 2015;22:317–328. doi:10.1016/j.chembiol.2015.02.005. 25794436

[76] Kaszaki J, Erces D, Varga G, Szabo A, Vecsei L, Boros M. Kynurenines and intestinal neurotransmission: the role of N-methyl-D-aspartate receptors. J Neural Transm (Vienna). 2012;119:211–223. doi:10.1007/s00702-011-0658-x. 21617892

[77] Stone TW, Darlington LG. The kynurenine pathway as a therapeutic target in cognitive and neurodegenerative disorders. Br J Pharmacol. 2013;169:1211–1227. doi:10.1111/bph.12230. 23647169 PMC3831703

[78] Saito K, Markey SP, Heyes MP. Effects of immune activation on quinolinic acid and neuroactive kynurenines in the mouse. Neuroscience. 1992;51:25–39. 1465184 10.1016/0306-4522(92)90467-g

[79] Fujigaki H, Yamamoto Y, Saito K. L-Tryptophan-kynurenine pathway enzymes are therapeutic target for neuropsychiatric diseases: focus on cell type differences. Neuropharmacology. 2017;112:264–274. doi:10.1016/j.neuropharm.2016.01.011. 26767951

[80] LeBlanc JG, Milani C, de Giori GS, Sesma F, van Sinderen D, Ventura M. Bacteria as vitamin suppliers to their host: a gut microbiota perspective. Curr Opin Biotechnol. 2013;24:160–168. doi:10.1016/j.copbio.2012.08.005. 22940212

[81] Said HM, Mohammed ZM. Intestinal absorption of water-soluble vitamins: an update. Curr Opin Gastroenterol. 2006;22:140–146. doi:10.1097/01.mog.0000203870.22706.52. 16462170

[82] Merli GJ, Fink J. Vitamin K and thrombosis. Vitam Horm. 2008;78:265–279. doi:10.1016/S0083-6729(07)00013-1. 18374199

[83] McMillan D, Perreault T, Watanabe M, Chance G, Askin DF, Hall J. Neonatal personnel in Canada. Paediatr Child Health. 1997;2:193–197. doi:10.1093/pch/2.3.193. 20098518 PMC2802585

[84] Allison AC. The possible role of vitamin K deficiency in the pathogenesis of Alzheimer’s disease and in augmenting brain damage associated with cardiovascular disease. Med Hypotheses. 2001;57:151–155. doi:10.1054/mehy.2001.1307. 11461163

[85] Da Silva FL, Coelho Cerqueira E, de Freitas MS, Goncalves DL, Costa LT, Follmer C. Vitamins K interact with N-terminus alpha-synuclein and modulate the protein fibrillization in vitro. Exploring the interaction between quinones and alpha-synuclein. Neurochem Int. 2013;62:103–112. doi:10.1016/j.neuint.2012.10.001. 23064431

[86] Soutif-Veillon A, Ferland G, Rolland Y, Presse N, Boucher K, Feart C, Annweiler C. Increased dietary vitamin K intake is associated with less severe subjective memory complaint among older adults. Maturitas. 2016;93:131–136. doi:10.1016/j.maturitas.2016.02.004. 26923488

[87] Hu J, Nie Y, Chen J, Zhang Y, Wang Z, Fan Q, Yan X. Gradual changes of gut microbiota in weaned miniature Piglets. Front Microbiol. 2016;7:1727. doi:10.3389/fmicb.2016.01727. 27853453 PMC5090779

[88] Langohr HD, Petruch F, Schroth G. Vitamin B 1, B 2 and B 6 deficiency in neurological disorders. J Neurol. 1981;225:95–108. 6164769 10.1007/BF00313323

[89] Finglas PM, Wright AJ, Wolfe CA, Hart DJ, Wright DM, Dainty JR. Is there more to folates than neural-tube defects? Proc Nutr Soc. 2003;62:591–598. doi:10.1079/PNS2003271. 14692594

[90] Owen RT. Folate augmentation of antidepressant response. Drugs Today (Barc). 2013;49:791–798. doi:10.1358/dot.2013.49.12.2086138. 24524097

[91] Pan LA, Martin P, Zimmer T, Segreti AM, Kassiff S, McKain BW, Baca CA, Rengasamy M, Hyland K, Walano N, et al. Neurometabolic disorders: potentially Treatable abnormalities in patients with treatment-refractory depression and suicidal behavior. Am J Psychiatry. 2017;174:42–50. doi:10.1176/appi.ajp.2016.15111500. 27523499 PMC10171090

[92] Agnew-Blais JC, Wassertheil-Smoller S, Kang JH, Hogan PE, Coker LH, Snetselaar LG, Smoller JW. Folate, vitamin B-6, and vitamin B-12 intake and mild cognitive impairment and probable dementia in the women’s health initiative memory study. J Acad Nutr Diet. 2015;115:231–241. doi:10.1016/j.jand.2014.07.006. 25201007 PMC4312724

[93] Douaud G, Refsum H, de Jager CA, Jacoby R, Nichols TE, Smith SM, Smith AD. Preventing Alzheimer’s disease-related gray matter atrophy by B-vitamin treatment. Proc Natl Acad Sci U S A. 2013;110:9523–9528. doi:10.1073/pnas.1301816110. 23690582 PMC3677457

[94] Andermann ML, Lowell BB. Toward a wiring diagram understanding of appetite control. Neuron. 2017;95:757–778. doi:10.1016/j.neuron.2017.06.014. 28817798 PMC5657399

[95] Brookes SJ, Spencer NJ, Costa M, Zagorodnyuk VP. Extrinsic primary afferent signalling in the gut. Nat Rev Gastroenterol Hepatol. 2013;10:286–296. doi:10.1038/nrgastro.2013.29. 23438947

[96] Gribble FM, Reimann F. Enteroendocrine cells: chemosensors in the intestinal epithelium. Annu Rev Physiol. 2016;78:277–299. doi:10.1146/annurev-physiol-021115-105439. 26442437

[97] Roth GS. Mechanisms of altered hormone-neurotransmitter action during aging: from receptors to calcium mobilization. Annu Rev Gerontol Geriatr. 1990;10:132–146. 1983283 10.1007/978-3-662-38445-9_8

[98] Kaelberer MM, Bohorquez DV. The now and then of gut-brain signaling. Brain Res. 2018;1693:192–196. doi:10.1016/j.brainres.2018.03.027. 29580839 PMC6003878

[99] Kaelberer MM, Buchanan KL, Klein ME, Barth BB, Montoya MM, Shen X, Bohórquez DV. A gut-brain neural circuit for nutrient sensory transduction. Science. 2018;361:eaat5236. doi:10.1126/science.aat5236. 10.1126/science.aat5236 PMC6417812 30237325

[100] Lauritzen KH, Morland C, Puchades M, Holm-Hansen S, Hagelin EM, Lauritzen F, Attramadal H, Storm-Mathisen J, Gjedde A, Bergersen LH. Lactate receptor sites link neurotransmission, neurovascular coupling, and brain energy metabolism. Cereb Cortex. 2014;24:2784–2795. doi:10.1093/cercor/bht136. 23696276

[101] McAllister MS, Krizanac-Bengez L, Macchia F, Naftalin RJ, Pedley KC, Mayberg MR, Marroni M, Leaman S, Stanness KA, Janigro D. Mechanisms of glucose transport at the blood-brain barrier: an in vitro study. Brain Res. 2001;904:20–30. doi:10.1016/s0006-8993(01)02418-0. 11516408

[102] Kastin AJ, Akerstrom V, Pan W. Interactions of glucagon-like peptide-1 (GLP-1) with the blood-brain barrier. J Mol Neurosci. 2002;18:7–14. doi:10.1385/JMN:18:1-2:07. 11931352

[103] Yamamoto H, Kishi T, Lee CE, Choi BJ, Fang H, Hollenberg AN, Drucker DJ, Elmquist JK. Glucagon-like peptide-1-responsive catecholamine neurons in the area postrema link peripheral glucagon-like peptide-1 with central autonomic control sites. J Neurosci. 2003;23:2939–2946. 12684481 10.1523/JNEUROSCI.23-07-02939.2003 PMC6742071

[104] Kieffer TJ, McIntosh CH, Pederson RA. Degradation of glucose-dependent insulinotropic polypeptide and truncated glucagon-like peptide 1 in vitro and in vivo by dipeptidyl peptidase IV. Endocrinology. 1995;136:3585–3596. doi:10.1210/endo.136.8.7628397. 7628397

[105] Neunlist M, Schemann M. Nutrient-induced changes in the phenotype and function of the enteric nervous system. J Physiol. 2014;592:2959–2965. doi:10.1113/jphysiol.2014.272948. 24907307 PMC4214652

[106] Abot A, Cani PD, Knauf C. Impact of intestinal peptides on the enteric nervous system: novel approaches to control glucose metabolism and food intake. Front Endocrinol (Lausanne). 2018;9:328. doi:10.3389/fendo.2018.00420. 29988396 PMC6023997

[107] Su Z, Alhadeff AL, Betley JN. Nutritive, post-ingestive signals are the primary regulators of AgRP neuron activity. Cell Rep. 2017;21:2724–2736. doi:10.1016/j.celrep.2017.11.036. 29212021 PMC5724395

[108] Dockray GJ. Enteroendocrine cell signalling via the vagus nerve. Curr Opin Pharmacol. 2013;13:954–958. doi:10.1016/j.coph.2013.09.007. 24064396

[109] Nohr MK, Egerod KL, Christiansen SH, Gille A, Offermanns S, Schwartz TW, Møller M. Expression of the short chain fatty acid receptor GPR41/FFAR3 in autonomic and somatic sensory ganglia. Neuroscience. 2015;290:126–137. doi:10.1016/j.neuroscience.2015.01.040. 25637492

[110] Poole DP, Godfrey C, Cattaruzza F, Cottrell GS, Kirkland JG, Pelayo JC, Bunnett NW, Corvera CU, et al. Expression and function of the bile acid receptor GpBAR1 (TGR5) in the murine enteric nervous system. Neurogastroenterol Motil. 2010;22(814–25):e227–8. doi:10.1111/j.1365-2982.2010.01487.x. PMC2891892 20236244

[111] Soret R, Chevalier J, De Coppet P, Poupeau G, Derkinderen P, Segain JP, Neunlist M. Short-chain fatty acids regulate the enteric neurons and control gastrointestinal motility in rats. Gastroenterology. 2010;138:1772–1782. doi:10.1053/j.gastro.2010.01.053. 20152836

[112] Vincent KM, Sharp JW, Raybould HE. Intestinal glucose-induced calcium-calmodulin kinase signaling in the gut-brain axis in awake rats. Neurogastroenterol Motil. 2011;23:e282–93. doi:10.1111/j.1365-2982.2011.01673.x. 21303432 PMC3101276

[113] Bohorquez DV, Shahid RA, Erdmann A, Kreger AM, Wang Y, Calakos N, Wang F, Liddle RA. Neuroepithelial circuit formed by innervation of sensory enteroendocrine cells. J Clin Invest. 2015;125:782–786. doi:10.1172/JCI78361. 25555217 PMC4319442

[114] Schretter CE, Vielmetter J, Bartos I, Marka Z, Marka S, Argade S, Mazmanian SK. A gut microbial factor modulates locomotor behaviour in *Drosophila*. Nature. 2018;563:402–406. doi:10.1038/s41586-018-0634-9. 30356215 PMC6237646

[115] Zhao HW, Yue YH, Han H, Chen XL, Lu YG, Zheng JM, Hou H-T, Lang X-M, He -L-L, Hu Q-L, et al. Effect of toll-like receptor 3 agonist poly I:C on intestinal mucosa and epithelial barrier function in mouse models of acute colitis. World J Gastroenterol. 2017;23:999–1009. doi:10.3748/wjg.v23.i6.999. 28246473 PMC5311109

[116] Mark KS, Miller DW. Increased permeability of primary cultured brain microvessel endothelial cell monolayers following TNF-alpha exposure. Life Sci. 1999;64:1941–1953. doi:10.1016/s0024-3205(99)00139-3. 10353592

[117] Thomsen MS, Birkelund S, Burkhart A, Stensballe A, Moos T. Synthesis and deposition of basement membrane proteins by primary brain capillary endothelial cells in a murine model of the blood-brain barrier. J Neurochem. 2017;140:741–754. doi:10.1111/jnc.13747. 27456748

[118] Perry VH. Stress primes microglia to the presence of systemic inflammation: implications for environmental influences on the brain. Brain Behav Immun. 2007;21:45–46. doi:10.1016/j.bbi.2006.08.004. 17011745

[119] Wu SC, Cao ZS, Chang KM, Juang JL. Intestinal microbial dysbiosis aggravates the progression of Alzheimer’s disease in *Drosophila*. Nat Commun. 2017;8:24. doi:10.1038/s41467-017-00040-6. 28634323 PMC5478647

[120] Williams SK, Fairless R, Maier O, Liermann PC, Pichi K, Fischer R, Eisel ULM, Kontermann R, Herrmann A, Weksler B, et al. Anti-TNFR1 targeting in humanized mice ameliorates disease in a model of multiple sclerosis. Sci Rep. 2018;8:13628. doi:10.1038/s41598-018-31957-7. 30206422 PMC6133964

[121] Becker L, Nguyen L, Gill J, Kulkarni S, Pasricha PJ, Habtezion A. Age-dependent shift in macrophage polarisation causes inflammation-mediated degeneration of enteric nervous system. Gut. 2018;67:827–836. doi:10.1136/gutjnl-2016-312940. 28228489 PMC5565713

[122] Rosenbaum C, Schick MA, Wollborn J, Heider A, Scholz CJ, Cecil A, Niesler B, Hirrlinger J, Walles H, Metzger M, et al. Activation of myenteric Glia during acute inflammation in vitro and in vivo. PLoS One. 2016;11:e0151335. doi:10.1371/journal.pone.0151335. 26964064 PMC4786261

[123] Alkahtani SH. The steroidal Na+/K+ ATPase inhibitor 3-[(R)-3-pyrrolidinyl]oxime derivative (3-R-POD) induces potent pro-apoptotic responses in colonic tumor cells. Anticancer Res. 2014;34:2967–2971. 24922661

[124] Kabouridis PS, Lasrado R, McCallum S, Chng SH, Snippert HJ, Clevers H, Pettersson S, Pachnis V. The gut microbiota keeps enteric glial cells on the move; prospective roles of the gut epithelium and immune system. Gut Microbes. 2015;6:398–403. doi:10.1080/19490976.2015.1109767. 26558327 PMC4826126

[125] Ibiza S, Garcia-Cassani B, Ribeiro H, Carvalho T, Almeida L, Marques R, Misic AM, Bartow-McKenney C, Larson DM, Pavan WJ, et al. Glial-cell-derived neuroregulators control type 3 innate lymphoid cells and gut defence. Nature. 2016;535:440–443. doi:10.1038/nature18644. 27409807 PMC4962913

[126] Jackerott M, Larsson LI. Immunocytochemical localization of the NPY/PYY Y1 receptor in enteric neurons, endothelial cells, and endocrine-like cells of the rat intestinal tract. J Histochem Cytochem. 1997;45:1643–1650. doi:10.1177/002215549704501207. 9389767

[127] Terry N, Margolis KG. Serotonergic mechanisms regulating the GI tract: experimental evidence and therapeutic relevance. Handb Exp Pharmacol. 2017;239:319–342. doi:10.1007/164_2016_103. 28035530 PMC5526216

[128] Takaki M, Branchek T, Tamir H, Gershon MD. Specific antagonism of enteric neural serotonin receptors by dipeptides of 5-hydroxytryptophan: evidence that serotonin is a mediator of slow synaptic excitation in the myenteric plexus. J Neurosci. 1985;5:1769–1780. 3874933 10.1523/JNEUROSCI.05-07-01769.1985 PMC6565118

[129] Williams DA, Zaidi SA, Zhang Y. 5-Hydroxy-2-(2-phenylethyl)chromone (5-HPEC): a novel non-nitrogenous ligand for 5-HT2B receptor. Bioorg Med Chem Lett. 2014;24:1489–1492. doi:10.1016/j.bmcl.2014.02.029. 24582985 PMC4003898

[130] De Vadder F, Grasset E, Manneras Holm L, Karsenty G, Macpherson AJ, Olofsson LE, Bäckhed F. Gut microbiota regulates maturation of the adult enteric nervous system via enteric serotonin networks. Proc Natl Acad Sci U S A. 2018;115:6458–6463. doi:10.1073/pnas.1720017115. 29866843 PMC6016808

[131] Xu Y, Yan J, Zhou P, Li J, Gao H, Xia Y, Wang Q. Neurotransmitter receptors and cognitive dysfunction in Alzheimer’s disease and Parkinson’s disease. Prog Neurobiol. 2012;97:1–13. doi:10.1016/j.pneurobio.2012.02.002. 22387368 PMC3371373

[132] Barajon I, Serrao G, Arnaboldi F, Opizzi E, Ripamonti G, Balsari A, Rumio C. Toll-like receptors 3, 4, and 7 are expressed in the enteric nervous system and dorsal root ganglia. J Histochem Cytochem. 2009;57:1013–1023. doi:10.1369/jhc.2009.953539. 19546475 PMC2762881

[133] Brun P, Giron MC, Qesari M, Porzionato A, Caputi V, Zoppellaro C, Banzato S, Grillo AR, Spagnol L, De Caro R, et al. Toll-like receptor 2 regulates intestinal inflammation by controlling integrity of the enteric nervous system. Gastroenterology. 2013;145:1323–1333. doi:10.1053/j.gastro.2013.08.047. 23994200

[134] Spielman LJ, Gibson DL, Klegeris A. Unhealthy gut, unhealthy brain: the role of the intestinal microbiota in neurodegenerative diseases. Neurochem Int. 2018;120:149–163. doi:10.1016/j.neuint.2018.08.005. 30114473

[135] Hyland NP, Cryan JF. A Gut Feeling about GABA: focus on GABA(B) Receptors. Front Pharmacol. 2010;1:124. doi:10.3389/fphar.2010.00124. 21833169 PMC3153004

[136] Ji XB, Hollocher TC. Reduction of nitrite to nitric oxide by enteric bacteria. Biochem Biophys Res Commun. 1988;157:106–108. doi:10.1016/s0006-291x(88)80018-4. 3058123

[137] Stanaszek PM, Snell JF, O’Neill JJ. Isolation, extraction, and measurement of acetylcholine from *Lactobacillus plantarum*. Appl Environ Microbiol. 1977;34:237–239. 907345 10.1128/aem.34.2.237-239.1977 PMC242629

[138] Tsavkelova EA, Botvinko IV, Kudrin VS, Oleskin AV. Detection of neurotransmitter amines in microorganisms with the use of high-performance liquid chromatography. Dokl Biochem. 2000;372:115–117. 10935181

[139] Özoğul F. Production of biogenic amines by *Morganella morganii*, *Klebsiella pneumoniae* and *Hafnia alvei* using a rapid HPLC method. Eur Food Res Technol. 2004;219:465–469. doi:10.1007/s00217-004-0988-0.

[140] Dinan TG, Stanton C, Cryan JF. Psychobiotics: a novel class of psychotropic. Biol Psychiatry. 2013;74:720–726. doi:10.1016/j.biopsych.2013.05.001. 23759244

[141] Bambury A, Sandhu K, Cryan JF, Dinan TG. Finding the needle in the haystack: systematic identification of psychobiotics. Br J Pharmacol. 2018;175:4430–4438. doi:10.1111/bph.14127. 29243233 PMC6255950

[142] Skelly DT, Hennessy E, Dansereau MA, Cunningham C. A systematic analysis of the peripheral and CNS effects of systemic LPS, IL-1beta, [corrected] TNF-alpha and IL-6 challenges in C57BL/6 mice. PLoS One. 2013;8:e69123. doi:10.1371/journal.pone.0069123. 23840908 PMC3698075

[143] Morton GJ, Meek TH, Schwartz MW. Neurobiology of food intake in health and disease. Nat Rev Neurosci. 2014;15:367–378. doi:10.1038/nrn3745. 24840801 PMC4076116

[144] Benakis C, Brea D, Caballero S, Faraco G, Moore J, Murphy M, Sita G, Racchumi G, Ling L, Pamer EG, et al. Commensal microbiota affects ischemic stroke outcome by regulating intestinal gammadelta T cells. Nat Med. 2016;22:516–523. doi:10.1038/nm.4068. 27019327 PMC4860105

[145] Banks WA. Brain meets body: the blood-brain barrier as an endocrine interface. Endocrinology. 2012;153:4111–4119. doi:10.1210/en.2012-1435. 22778219 PMC3423627

[146] Louveau A, Herz J, Alme MN, Salvador AF, Dong MQ, Viar KE, Herod SG, Knopp J, Setliff JC, Lupi AL, et al. CNS lymphatic drainage and neuroinflammation are regulated by meningeal lymphatic vasculature. Nat Neurosci. 2018;21:1380–1391. doi:10.1038/s41593-018-0227-9. 30224810 PMC6214619

[147] Herisson F, Frodermann V, Courties G, Rohde D, Sun Y, Vandoorne K, Wojtkiewicz GR, Masson GS, Vinegoni C, Kim J, et al. Direct vascular channels connect skull bone marrow and the brain surface enabling myeloid cell migration. Nat Neurosci. 2018;21:1209–1217. doi:10.1038/s41593-018-0213-2. 30150661 PMC6148759

[148] Nakamura YK, Janowitz C, Metea C, Asquith M, Karstens L, Rosenbaum JT, Lin P. Short chain fatty acids ameliorate immune-mediated uveitis partially by altering migration of lymphocytes from the intestine. Sci Rep. 2017;7:11745. doi:10.1038/s41598-017-12163-3. 28924192 PMC5603543

[149] Braak H, Ghebremedhin E, Rub U, Bratzke H, Del Tredici K. Stages in the development of Parkinson’s disease-related pathology. Cell Tissue Res. 2004;318:121–134. doi:10.1007/s00441-004-0956-9. 15338272

[150] Peelaerts W, Bousset L, Van der Perren A, Moskalyuk A, Pulizzi R, Giugliano M, Van Den Haute C, Melki R, Baekelandt V. alpha-Synuclein strains cause distinct synucleinopathies after local and systemic administration. Nature. 2015;522:340–344. doi:10.1038/nature14547. 26061766

[151] Breen DP, Halliday GM, Lang AE. Gut-brain axis and the spread of alpha-synuclein pathology: vagal highway or dead end? Mov Disord. 2019;34:307–316. doi:10.1002/mds.27556. 30653258

[152] Pan-Montojo F, Anichtchik O, Dening Y, Knels L, Pursche S, Jung R, Jackson S, Gille G, Spillantini MG, Reichmann H, et al. Progression of Parkinson’s disease pathology is reproduced by intragastric administration of rotenone in mice. PLoS One. 2010;5:e8762. doi:10.1371/journal.pone.0008762. 20098733 PMC2808242

[153] Pan-Montojo F, Schwarz M, Winkler C, Arnhold M, O’Sullivan GA, Pal A, Said J, Marsico G, Verbavatz JM, Rodrigo-Angulo M, et al. Environmental toxins trigger PD-like progression via increased alpha-synuclein release from enteric neurons in mice. Sci Rep. 2012;2:898. doi:10.1038/srep00386. 23205266 PMC3510466

[154] Holmqvist S, Chutna O, Bousset L, Aldrin-Kirk P, Li W, Bjorklund T, Wang Z-Y, Roybon L, Melki R, Li J-Y. Direct evidence of Parkinson pathology spread from the gastrointestinal tract to the brain in rats. Acta Neuropathol. 2014;128:805–820. doi:10.1007/s00401-014-1343-6. 25296989

[155] Uemura N, Yagi H, Uemura MT, Hatanaka Y, Yamakado H, Takahashi R. Inoculation of alpha-synuclein preformed fibrils into the mouse gastrointestinal tract induces Lewy body-like aggregates in the brainstem via the vagus nerve. Mol Neurodegener. 2018;13:21. doi:10.1186/s13024-018-0257-5. 29751824 PMC5948849

[156] Chandra R, Hiniker A, Kuo YM, Nussbaum RL, Liddle RA. alpha-Synuclein in gut endocrine cells and its implications for Parkinson’s disease. JCI Insight. 2017;2:e92295. doi:10.1172/jci.insight.92295. 10.1172/jci.insight.92295 PMC5470880 28614796

[157] Rey NL, Petit GH, Bousset L, Melki R, Brundin P. Transfer of human alpha-synuclein from the olfactory bulb to interconnected brain regions in mice. Acta Neuropathol. 2013;126:555–573. doi:10.1007/s00401-013-1160-3. 23925565 PMC3789892

[158] Svensson E, Horvath-Puho E, Thomsen RW, Djurhuus JC, Pedersen L, Borghammer P, Sørensen HT. Vagotomy and subsequent risk of Parkinson’s disease. Ann Neurol. 2015;78:522–529. doi:10.1002/ana.24448. 26031848

[159] Liu B, Fang F, Pedersen NL, Tillander A, Ludvigsson JF, Ekbom A, Svenningsson P, Chen H, Wirdefeldt K. Vagotomy and Parkinson disease: a Swedish register-based matched-cohort study. Neurology. 2017;88:1996–2002. doi:10.1212/WNL.0000000000003961. 28446653 PMC5440238

[160] Lin SY, Lin CL, Wang IK, Lin CC, Lin CH, Hsu WH, Kao C-H. Dementia and vagotomy in Taiwan: a population-based cohort study. BMJ Open. 2018;8:e019582. doi:10.1136/bmjopen-2017-019582. PMC5884346 29602843

[161] Guinane CM, Tadrous A, Fouhy F, Ryan CA, Dempsey EM, Murphy B, Andrews E, Cotter PD, Stanton C, Ross RP. Microbial composition of human appendices from patients following appendectomy. MBio. 2013;4:e00366-12. doi:10.1128/mBio.00366-12. 10.1128/mBio.00366-12 PMC3551545 23322636

[162] Gray MT, Munoz DG, Gray DA, Schlossmacher MG, Woulfe JM. Alpha-synuclein in the appendiceal mucosa of neurologically intact subjects. Mov Disord. 2014;29:991–998. doi:10.1002/mds.25779. 24352892

[163] Mendes A, Goncalves A, Vila-Cha N, Moreira I, Fernandes J, Damasio J, Teixeira-Pinto A, Taipa R, Lima AB, Cavaco S. Appendectomy may delay Parkinson’s disease Onset. Mov Disord. 2015;30:1404–1407. doi:10.1002/mds.26311. 26228745

[164] Yilmaz R, Bayram E, Ulukan C, Altinok MK, Akbostanci MC. Appendectomy history is not related to Parkinson’s Disease. J Parkinsons Dis. 2017;7:347–352. doi:10.3233/JPD-171071. 28387683

[165] Marras C, Lang AE, Austin PC, Lau C, Urbach DR. Appendectomy in mid and later life and risk of Parkinson’s disease: a population-based study. Mov Disord. 2016;31:1243–1247. doi:10.1002/mds.26670. 27241338

[166] Svensson E, Horvath-Puho E, Stokholm MG, Sorensen HT, Henderson VW, Borghammer P. Appendectomy and risk of Parkinson’s disease: a nationwide cohort study with more than 10 years of follow-up. Mov Disord. 2016;31:1918–1922. doi:10.1002/mds.26761. 27621223

[167] Chen SG, Stribinskis V, Rane MJ, Demuth DR, Gozal E, Roberts AM, Jagadapillai R, Liu R, Choe K, Shivakumar B, et al. Exposure to the functional bacterial amyloid protein curli enhances alpha-synuclein aggregation in aged fischer 344 rats and *Caenorhabditis elegans*. Sci Rep. 2016;6:34477. doi:10.1038/srep34477. 27708338 PMC5052651

[168] Gibson GR, Roberfroid MB. Dietary modulation of the human colonic microbiota: introducing the concept of prebiotics. J Nutr. 1995;125:1401–1412. doi:10.1093/jn/125.6.1401. 7782892

[169] Roberfroid M. Probiotics and prebiotics: why should the medical community pay attention? Drug Discov Today. 2003;8:1107–1108. 14678734 10.1016/s1359-6446(03)02844-7

[170] Iannitti T, Palmieri B. Therapeutical use of probiotic formulations in clinical practice. Clin Nutr. 2010;29:701–725. doi:10.1016/j.clnu.2010.05.004. 20576332 PMC7172412

[171] Bindels LB, Delzenne NM, Cani PD, Walter J. Towards a more comprehensive concept for prebiotics. Nat Rev Gastroenterol Hepatol. 2015;12:303–310. doi:10.1038/nrgastro.2015.47. 25824997

[172] Savignac HM, Corona G, Mills H, Chen L, Spencer JP, Tzortzis G, Burnet PWJ. Prebiotic feeding elevates central brain derived neurotrophic factor, N-methyl-D-aspartate receptor subunits and D-serine. Neurochem Int. 2013;63:756–764. doi:10.1016/j.neuint.2013.10.006. 24140431 PMC3858812

[173] Kao A-C-C, Chan KW, Anthony DC, Lennox BR, Burnet PW. Prebiotic reduction of brain histone deacetylase (HDAC) activity and olanzapine-mediated weight gain in rats, are acetate independent. Neuropharmacology. 2019;150:184–191. doi:10.1016/j.neuropharm.2019.02.014. 30763656

[174] Mika A, Gaffney M, Roller R, Hills A, Bouchet CA, Hulen KA, Thompson RS, Chichlowski M, Berg BM, Fleshner M. Feeding the developing brain: juvenile rats fed diet rich in prebiotics and bioactive milk fractions exhibit reduced anxiety-related behavior and modified gene expression in emotion circuits. Neurosci Lett. 2018;677:103–109. doi:10.1016/j.neulet.2018.01.052. 29409860

[175] de Cossío LF, Fourrier C, Sauvant J, Everard A, Capuron L, Cani PD, Layé S, Castanon N. Impact of prebiotics on metabolic and behavioral alterations in a mouse model of metabolic syndrome. Brain Behav Immun. 2017;64:33–49. doi:10.1016/j.bbi.2016.12.022. 28027925

[176] Burokas A, Arboleya S, Moloney RD, Peterson VL, Murphy K, Clarke G, Stanton C, Dinan TG, Cryan JF. Targeting the microbiota-gut-brain axis: prebiotics have anxiolytic and antidepressant-like effects and reverse the impact of chronic stress in mice. Biol Psychiatry. 2017;82:472–487. doi:10.1016/j.biopsych.2016.12.031. 28242013

[177] Crumeyrolle-Arias M, Jaglin M, Bruneau A, Vancassel S, Cardona A, Dauge V, Naudon L, Rabot S. Absence of the gut microbiota enhances anxiety-like behavior and neuroendocrine response to acute stress in rats. Psychoneuroendocrinology. 2014;42:207–217. doi:10.1016/j.psyneuen.2014.01.014. 24636517

[178] Sudo N, Chida Y, Aiba Y, Sonoda J, Oyama N, Yu XN, Kubo C, Koga Y. Postnatal microbial colonization programs the hypothalamic-pituitary-adrenal system for stress response in mice. J Physiol. 2004;558:263–275. doi:10.1113/jphysiol.2004.063388. 15133062 PMC1664925

[179] Kao AC, Spitzer S, Anthony DC, Lennox B, Burnet PWJ. Prebiotic attenuation of olanzapine-induced weight gain in rats: analysis of central and peripheral biomarkers and gut microbiota. Transl Psychiatry. 2018;8:66. doi:10.1038/s41398-018-0116-8. 29540664 PMC5852210

[180] Schmidt K, Cowen PJ, Harmer CJ, Tzortzis G, Errington S, Burnet PW. Prebiotic intake reduces the waking cortisol response and alters emotional bias in healthy volunteers. Psychopharmacology (Berl). 2015;232:1793–1801. doi:10.1007/s00213-014-3810-0. 25449699 PMC4410136

[181] van Den Berg JP, Westerbeek EA, Broring-Starre T, Garssen J, van Elburg RM. Neurodevelopment of preterm infants at 24 months after neonatal supplementation of a prebiotic mix: a randomized trial. J Pediatr Gastroenterol Nutr. 2016;63:270–276. 26859091 10.1097/MPG.0000000000001148

[182] Romo-Araiza A, Gutierrez-Salmean G, Galvan EJ, Hernandez-Frausto M, Herrera-Lopez G, Romo-Parra H, García-Contreras V, Fernández-Presas AM, Jasso-Chávez R, Borlongan CV, et al. Probiotics and prebiotics as a therapeutic strategy to improve memory in a model of middle-aged rats. Front Aging Neurosci. 2018;10:416. doi:10.3389/fnagi.2018.00416. 30618722 PMC6305305

[183] Rajkumar H, Kumar M, Das N, Kumar SN, Challa HR, Nagpal R. Effect of probiotic *Lactobacillus salivarius* UBL S22 and prebiotic fructo-oligosaccharide on Serum Lipids, inflammatory markers, insulin sensitivity, and gut bacteria in healthy young volunteers: a randomized controlled single-blind pilot study. J Cardiovasc Pharmacol Ther. 2015;20:289–298. doi:10.1177/1074248414555004. 25331262

[184] De Paula JA, Carmuega E, Weill R. Effect of the ingestion of a symbiotic yogurt on the bowel habits of women with functional constipation. Acta Gastroenterol Latinoam. 2008;38:16–25. 18533353

[185] FAO/WHO. Expert consultation on evaluation of health and nutritional properties of probiotics in food including powder milk with live lactic acid bacteria. Córdoba (Argentina) 10 2001:1–4.

[186] Kunes J, Prazienkova V, Popelova A, Mikulaskova B, Zemenova J, Maletinska L. Prolactin-releasing peptide: a new tool for obesity treatment. J Endocrinol. 2016;230:R51–8. doi:10.1530/JOE-16-0046. 27418033

[187] Cani PD, de Vos WM. Next-generation beneficial microbes: the case of *Akkermansia muciniphila*. Front Microbiol. 2017;8:1765. doi:10.3389/fmicb.2017.01765. 29018410 PMC5614963

[188] Breyner NM, Michon C, de Sousa CS, Vilas Boas PB, Chain F, Azevedo VA, Langella P, Chatel JM. Microbial Anti-Inflammatory Molecule (MAM) from *Faecalibacterium prausnitzii* shows a protective effect on DNBS and DSS-induced colitis model in mice through inhibition of NF-kappaB pathway. Front Microbiol. 2017;8:114. doi:10.3389/fmicb.2017.00114. 28203226 PMC5285381

[189] Hamady ZZ, Scott N, Farrar MD, Lodge JP, Holland KT, Whitehead T, Carding SR. Xylan-regulated delivery of human keratinocyte growth factor-2 to the inflamed colon by the human anaerobic commensal bacterium *Bacteroides ovatus*. Gut. 2010;59:461–469. doi:10.1136/gut.2008.176131. 19736360

[190] Saarela MH. Safety aspects of next generation probiotics. Curr Opin Food Sci. 2019;30:8–13. doi:10.1016/j.cofs.2018.09.001.

[191] Aguilar-Toala JE, Astiazaran-Garcia H, Estrada-Montoya MC, Garcia HS, Vallejo-Cordoba B, Gonzalez-Cordova AF, Hernández-Mendoza A. Modulatory effect of the intracellular content of *Lactobacillus casei* CRL 431 against the aflatoxin B1-Induced oxidative stress in rats. Probiotics Antimicrob Proteins. 2019;11:470–477. doi:10.1007/s12602-018-9433-8. 10.1007/s12602-018-9433-8 29862461

[192] Taverniti V, Guglielmetti S. The immunomodulatory properties of probiotic microorganisms beyond their viability (ghost probiotics: proposal of paraprobiotic concept). Genes Nutr. 2011;6:261–274. doi:10.1007/s12263-011-0218-x. 21499799 PMC3145061

[193] Tsilingiri K, Rescigno M. Postbiotics: what else? Benef Microbes. 2013;4:101–107. doi:10.3920/BM2012.0046. 23271068

[194] Phillips JGP. The treatment of melancholia by the lactic acid bacillus. J Mental Sci. 1910;56:422–430. doi:10.1192/bjp.56.234.422.

[195] Messaoudi M, Lalonde R, Violle N, Javelot H, Desor D, Nejdi A, Bisson J-F, Rougeot C, Pichelin M, Cazaubiel M, et al. Assessment of psychotropic-like properties of a probiotic formulation (*Lactobacillus helveticus* R0052 and *Bifidobacterium longum* R0175) in rats and human subjects. Br J Nutr. 2011;105:755–764. doi:10.1017/S0007114510004319. 20974015

[196] Kato-Kataoka A, Nishida K, Takada M, Suda K, Kawai M, Shimizu K, Kushiro A, Hoshi R, Watanabe O, Igarashi T, et al. Fermented milk containing *Lactobacillus casei* strain Shirota prevents the onset of physical symptoms in medical students under academic examination stress. Benef Microbes. 2016;7:153–156. doi:10.3920/BM2015.0100. 26689231

[197] Slykerman RF, Hood F, Wickens K, Thompson JMD, Barthow C, Murphy R, Kang J, Rowden J, Stone P, Crane J, et al. Effect of *Lactobacillus rhamnosus* HN001 in Pregnancy on Postpartum Symptoms of Depression and Anxiety: a Randomised Double-blind Placebo-controlled Trial. EBioMedicine. 2017;24:159–165. doi:10.1016/j.ebiom.2017.09.013. 28943228 PMC5652021

[198] Lu Z, Zhang W, Zhang N, Jiang J, Luo Q, Qiu Y. The expression of glutamate transporters in chest compression-induced audiogenic epilepsy: a comparative study. Neurol Res. 2008;30:915–919. doi:10.1179/174313208X327964. 18671901

[199] Sarkar A, Lehto SM, Harty S, Dinan TG, Cryan JF, Burnet PWJ. Psychobiotics and the Manipulation of Bacteria-Gut-Brain Signals. Trends Neurosci. 2016;39:763–781. doi:10.1016/j.tins.2016.09.002. 27793434 PMC5102282

[200] Cassani E, Privitera G, Pezzoli G, Pusani C, Madio C, Iorio L, Barichella M. Use of probiotics for the treatment of constipation in Parkinson’s disease patients. Minerva Gastroenterol Dietol. 2011;57:117–121. 21587143

[201] Barichella M, Pacchetti C, Bolliri C, Cassani E, Iorio L, Pusani C, Pinelli G, Privitera G, Cesari I, Faierman SA, et al. Probiotics and prebiotic fiber for constipation associated with Parkinson disease: an RCT. Neurology. 2016;87:1274–1280. doi:10.1212/WNL.0000000000003127. 27543643

[202] Tamtaji OR, Taghizadeh M, Daneshvar Kakhaki R, Kouchaki E, Bahmani F, Borzabadi S, Oryan S, Mafi A, Asemi Z. Clinical and metabolic response to probiotic administration in people with Parkinson’s disease: a randomized, double-blind, placebo-controlled trial. Clin Nutr. 2019;38:1031–1035. doi:10.1016/j.clnu.2018.05.018. 29891223

[203] Nimgampalle M, Kuna Y. Anti-Alzheimer properties of probiotic, *Lactobacillus plantarum* MTCC 1325 in Alzheimer’s Disease induced Albino rats. J Clin Diagn Res. 2017;11:KC01–KC5. doi:10.7860/JCDR/2017/26106.10428. PMC5620801 28969160

[204] Athari Nik Azm S, Djazayeri A, Safa M, Azami K, Ahmadvand B, Sabbaghziarani F, Sharifzadeh M, Vafa M. Lactobacilli and bifidobacteria ameliorate memory and learning deficits and oxidative stress in beta-amyloid (1-42) injected rats. Appl Physiol Nutr Metab. 2018;43:718–726. doi:10.1139/apnm-2017-0648. 29462572

[205] Bonfili L, Cecarini V, Berardi S, Scarpona S, Suchodolski JS, Nasuti C, Fiorini D, Boarelli MC, Rossi G, Eleuteri AM. Microbiota modulation counteracts Alzheimer’s disease progression influencing neuronal proteolysis and gut hormones plasma levels. Sci Rep. 2017;7:2426. doi:10.1038/s41598-017-02587-2. 28546539 PMC5445077

[206] Leblhuber F, Steiner K, Schuetz B, Fuchs D, Gostner JM. Probiotic Supplementation in patients with Alzheimer’s Dementia - an explorative intervention study. Curr Alzheimer Res. 2018;15:1106–1113. doi:10.2174/1389200219666180813144834. 30101706 PMC6340155

[207] Kobayashi Y, Kinoshita T, Matsumoto A, Yoshino K, Saito I, Xiao JZ. *Bifidobacterium breve* A1 supplementation improved cognitive decline in older adults with mild cognitive impairment: an open-label, single-arm study. J Prev Alzheimers Dis. 2019;6:70–75. doi:10.14283/jpad.2018.32.30569089

[208] Akbari E, Asemi Z, Daneshvar Kakhaki R, Bahmani F, Kouchaki E, Tamtaji OR, Hamidi GA, Salami M. Effect of probiotic supplementation on cognitive function and metabolic status in Alzheimer’s disease: a randomized, double-blind and controlled trial. Front Aging Neurosci. 2016;8:256. doi:10.3389/fnagi.2016.00256. 27891089 PMC5105117

[209] Tamtaji OR, Heidari-Soureshjani R, Mirhosseini N, Kouchaki E, Bahmani F, Aghadavod E, Tajabadi-Ebrahimi M, Asemi Z. Probiotic and selenium co-supplementation, and the effects on clinical, metabolic and genetic status in Alzheimer’s disease: a randomized, double-blind, controlled trial. Clin Nutr. 2018. doi:10.1016/j.clnu.2018.11.034. 30642737

[210] Staley C, Khoruts A, Sadowsky MJ. Contemporary applications of fecal microbiota transplantation to treat intestinal diseases in humans. Arch Med Res. 2017;48:766–773. doi:10.1016/j.arcmed.2017.11.006. 29183720

[211] Zhang F, Luo W, Shi Y, Fan Z, Ji G. Should we standardize the 1,700-year-old fecal microbiota transplantation? Am J Gastroenterol. 2012;107:1755. author reply p −6. doi:10.1038/ajg.2012.251. 23160295

[212] Bakken JS. Fecal bacteriotherapy for recurrent *Clostridium difficile infection*. Anaerobe. 2009;15:285–289. doi:10.1016/j.anaerobe.2009.09.007. 19778623

[213] Moayyedi P, Surette MG, Kim PT, Libertucci J, Wolfe M, Onischi C, Armstrong D, Marshall JK, Kassam Z, Reinisch W, et al. Fecal microbiota transplantation induces remission in patients with active ulcerative colitis in a randomized controlled trial. Gastroenterology. 2015;149:102–9 e6. doi:10.1053/j.gastro.2015.04.001. 25857665

[214] Schmulson M, Bashashati M. Fecal microbiota transfer for bowel disorders: efficacy or hype? Curr Opin Pharmacol. 2018;43:72–80. doi:10.1016/j.coph.2018.08.012. 30218939

[215] Bercik P, Denou E, Collins J, Jackson W, Lu J, Jury J, Deng Y, Blennerhassett P, Macri J, McCoy KD, et al. The intestinal microbiota affect central levels of brain-derived neurotropic factor and behavior in mice. Gastroenterology. 2011;141(599–609):e1–3. doi:10.1053/j.gastro.2011.04.052. 21683077

[216] Zheng P, Zeng B, Zhou C, Liu M, Fang Z, Xu X, Zeng L, Chen J, Fan S, Du X, et al. Gut microbiome remodeling induces depressive-like behaviors through a pathway mediated by the host’s metabolism. Mol Psychiatry. 2016;21:786–796. doi:10.1038/mp.2016.44. 27067014

[217] Kelly JR, Borre Y, OB C, Patterson E, El Aidy S, Deane J, Kennedy PJ, Beers S, Scott K, Moloney G, et al. Transferring the blues: depression-associated gut microbiota induces neurobehavioural changes in the rat. J Psychiatr Res. 2016;82:109–118. doi:10.1016/j.jpsychires.2016.07.019. 27491067

[218] McFarlane HG, Kusek GK, Yang M, Phoenix JL, Bolivar VJ, Crawley JN. Autism-like behavioral phenotypes in BTBR T+tf/J mice. Genes Brain Behav. 2008;7:152–163. doi:10.1111/j.1601-183X.2007.00330.x. 17559418

[219] Schwarcz R, Bruno JP, Muchowski PJ, Wu HQ. Kynurenines in the mammalian brain: when physiology meets pathology. Nat Rev Neurosci. 2012;13:465–477. doi:10.1038/nrn3257. 22678511 PMC3681811

[220] Sun MF, Zhu YL, Zhou ZL, Jia XB, Xu YD, Yang Q, Cui C, Shen Y-Q. Neuroprotective effects of fecal microbiota transplantation on MPTP-induced Parkinson’s disease mice: gut microbiota, glial reaction and TLR4/TNF-alpha signaling pathway. Brain Behav Immun. 2018;70:48–60. doi:10.1016/j.bbi.2018.02.005. 29471030

[221] Ananthaswamy A Faecal transplant eases symptoms of Parkinson’s disease. Elsevier, 2011.

[222] Woodworth MH, Carpentieri C, Sitchenko KL, Kraft CS. Challenges in fecal donor selection and screening for fecal microbiota transplantation: a review. Gut Microbes. 2017;8:225–237. doi:10.1080/19490976.2017.1286006. 28129018 PMC5479407

[223] Smith P, Willemsen D, Popkes M, Metge F, Gandiwa E, Reichard M, Valenzano DR. Regulation of life span by the gut microbiota in the short-lived African turquoise killifish. Elife. 2017;6:e27014. doi:10.7554/eLife.27014. 10.7554/eLife.27014 PMC5566455 28826469

